# SEC14L3 knockdown inhibited clear cell renal cell carcinoma proliferation, metastasis and sunitinib resistance through an SEC14L3/RPS3/NFκB positive feedback loop

**DOI:** 10.1186/s13046-024-03206-5

**Published:** 2024-10-19

**Authors:** Ziming Jiang, Guangcan Yang, Guangchun Wang, Jiayi Wan, Yifan Zhang, Wei Song, Houliang Zhang, Jinliang Ni, Haipeng Zhang, Ming Luo, Keyi Wang, Bo Peng

**Affiliations:** 1 Department of Urology, Shanghai Tenth People’s Hospital, Tongji University School of Medicine, Shanghai, 200072 China; 2 Department of Urology, Zhongshan Hospital, Fudan University, Shanghai, 200032 China; 3 Department of Nephrology, The First Affiliated Hospital of Zhengzhou University, Zhengzhou, 450000 China

**Keywords:** SEC14L3, RPS3, NFκB, Nanoparticles, Clear cell renal cell carcinoma, Tumor suppressor

## Abstract

**Background:**

Clear cell renal cell carcinoma (ccRCC) arises from the renal parenchymal epithelium and is the predominant malignant entity of renal cancer, exhibiting increasing incidence and mortality rates over time. SEC14-like 3 (SEC14L3) has emerged as a compelling target for cancer intervention; nevertheless, the precise clinical implications and molecular underpinnings of SEC14L3 in ccRCC remain elusive.

**Methods:**

By leveraging clinical data and data from the TCGA-ccRCC and GEO datasets, we investigated the association between SEC14L3 expression levels and overall survival rates in ccRCC patients. The biological role and mechanism of SEC14L3 in ccRCC were investigated via in vivo and in vitro experiments. Moreover, siRNA-SEC14L3@PDA@MUC12 nanoparticles (SSPM-NPs) were synthesized and assessed for their therapeutic potential against SEC14L3 through in vivo and in vitro assays.

**Results:**

Our investigation revealed upregulated SEC14L3 expression in ccRCC tissues, and exogenous downregulation of SEC14L3 robustly suppressed the malignant traits of ccRCC cells. Mechanistically, knocking down SEC14L3 facilitated the ubiquitination-mediated degradation of ribosomal protein S3 (RPS3) and augmented IκBα accumulation in ccRCC. This concerted action thwarted the nuclear translocation of P65, thereby abrogating the activation of the nuclear factor kappa B (NFκB) signaling pathway and impeding ccRCC cell proliferation and metastasis. Furthermore, diminished SEC14L3 levels exerted a suppressive effect on NFKB1 expression within the NFκB signaling cascade. NFKB1 functions as a transcriptional regulator capable of binding to the SEC14L3 enhancer and promoter, thereby promoting SEC14L3 expression. Consequently, the inhibition of SEC14L3 expression was further potentiated, thus forming a positive feedback loop. Additionally, we observed that downregulation of SEC14L3 significantly increased the sensitivity of ccRCC cells to sunitinib. The evaluation of SSPM-NPs nanotherapy highlighted its effectiveness in combination with sunitinib for inhibiting ccRCC growth.

**Conclusion:**

Our findings not only underscore the promise of SEC14L3 as a therapeutic target but also unveil an SEC14L3/RPS3/NFκB positive feedback loop that curtails ccRCC progression. Modulating SEC14L3 expression to engage this positive feedback loop might herald novel avenues for ccRCC treatment.

**Supplementary Information:**

The online version contains supplementary material available at 10.1186/s13046-024-03206-5.

## Introduction

Renal cell carcinoma (RCC) constitutes more than 90% of kidney cancer cases, with clear cell renal cell carcinoma (ccRCC) representing approximately 80% of RCC cases [1, 2]. Global 2020 statistics reveal over 430,000 new RCC cases across 185 countries, culminating in nearly 180,000 fatalities [3]. Radical surgery remains the primary treatment for early-stage ccRCC patients [4]. However, approximately 35% of patients present with metastatic disease at diagnosis [5], with approximately half developing metastatic lesions postoperatively [6]. The 5-year survival rate for advanced or metastatic disease is only 12% [7], highlighting the need to explore molecular mechanisms and therapeutic targets.

The SEC14-like 3 (SEC14L3) protein, part of the SEC14-like protein family, primarily acts as a phosphatidylinositol transfer protein, facilitating phosphatidylinositol and phosphatidylcholine exchange between membranes [8]. Structurally, it contains an SEC14 domain at its N-terminus and a Golgi dynamics (GOLD) domain at its C-terminus [9]. The GOLD domain shares sequence homology with the luminal domain of the p24 family protein KE8E4.6 in *Caenorhabditis elegans*, suggesting its involvement in protein-protein interactions [10]. SEC14L3 regulates lipid metabolism and phosphoinositide signaling pathways, responding to extracellular cues and modulating intracellular signaling [11–14]. Notably, current research reveals the involvement of SEC14L3, in conjunction with vascular endothelial growth factor, in regulating the migration of vascular endothelial and vein progenitor cells, thereby contributing to angiogenesis [15]. Additionally, emerging evidence implicates SEC14L3 in the progression of lung and breast cancers [16, 17], underscoring its potential significance in malignancy. However, further investigation is warranted to elucidate its role in RCC.

Nuclear factor kappa B (NFκB) is a multifaceted regulatory factor and a pivotal transcription factor involved in inflammation, immune responses, and carcinogenesis, impacting human cancer initiation and progression [18, 19]. Its aberrant activation in tumor cells promotes proliferation, inhibits apoptosis, modulates angiogenesis, alters metabolism, and engenders resistance to therapeutic agents [20, 21]. Ribosomal protein S3 (RPS3) is a component of the eukaryotic ribosomal 40 S subunit and is crucial for ribosome translation initiation [22]. RPS3, which acts as a non-Rel subunit in the NFκB complex, RPS3 directly interacts with NFκB P65, the facilitating transcriptional activation of NFκB-driven genes [23, 24]. In quiescent cells, RPS3 engages with the cytoplasmic NFκB P65-P50-IκBα complex [23, 24]. Under stimulation by factors such as tumor necrosis factor-alpha or lipopolysaccharide, the canonical NFκB pathway is activated, leading to NFκB Rel complex translocation alongside RPS3 to the nucleus [25]. RPS3 enhances NFκB activation, participating in physiological processes including DNA repair, apoptosis, and radioresistance [25–28]. Despite the understanding of the role of RPS3, its role in kidney cancer progression remains largely unexplored.

In this study, we found that elevated SEC14L3 in ccRCC correlates with poor prognosis. SEC14L3 knockdown inhibited ccRCC cell proliferation and metastasis by promoting RPS3 ubiquitination-mediated degradation and IκBα accumulation, blocking P65 nuclear translocation and NFκB pathway activation. Reduced NFKB1 in NFκB impedes SEC14L3 promoter and enhancer activation, forming a positive feedback loop that enhances tumor suppression. Finally, given the role of NFκB in enhancing sunitinib resistance [29, 30], we found that downregulation of SEC14L3 enhances the sensitivity of ccRCC cells to sunitinib treatment.

## Materials and methods

### Human specimens and cell cultures

All samples were obtained with informed consent from patients at the Department of Urology, Shanghai Tenth People’s Hospital. Fresh tissues obtained postoperatively were immediately preserved in liquid nitrogen. None of the patients received neoadjuvant chemotherapy or radiotherapy. The human ccRCC cell lines, OSRC-2 (RRID: CVCL_1626), ACHN (RRID: CVCL_1067), 786-O (RRID: CVCL_1051) and A498 (RRID: CVCL_1056) and the normal renal tubular epithelial cell line (HK-2) (RRID: CVCL_0302), were purchased from the Cell Bank of the Chinese Academy of Sciences (Shanghai, China). ACHN cells cultured in MEM medium (Gibco, Waltham, MA, USA), other cell lines were cultured in 1640 medium (Cytiva, Marlborough, MA, USA) in a humidified incubator with 5% CO_2_ at 37 °C. All culture medium supplemented with 10% fetal bovine serum (PAN Biotech, Aidenbach, Germany), 100 IU/mL penicillin, and 100 µg/mL streptomycin (Gibco, Waltham, MA, USA). The above cell lines were stored at -80 °C using CELL SAVING reagent (NCM, Suzhou, China).

### Quantitative real-time PCR (qRT‒PCR)

Total RNA was extracted using TRIzol reagent (Invitrogen, USA) according to the manufacturer’s instructions. Total RNA was reverse transcribed into cDNA using Prime Script RT Master Mix (RR036A; Takara). Subsequently, qRT‒PCR was performed using SYBR Green PCR Master Mix (Application Takara, Otsu, Japan) on a QuantStudio Dx/1 (Thermofishe, USA). The relative expression levels of each gene were calculated using the 2^−ΔΔCt^ method, with GAPDH serving as the internal reference control. The sequences of primers used in this study are listed in Table S1.

### Cell transfection

A small interfering RNA (siRNA) targeting SEC14L3 was synthesized by RiboBio Co., Ltd. (Guangzhou, China). SEC14L3 and NFKB1 knockdown lentiviruses were synthesized by Genechem Co., Ltd. (Shanghai, China). Transfection was performed using transfection reagent A (Genechem, Shanghai, China). The transfection efficiency was validated using qRT‒PCR and immunoblotting. The sequences of the siRNAs and lentiviruses used are listed in Table S2.

### Cell counting Kit-8 (CCK-8)

Pre-treated or transfected cells were seeded at a density of 1,000 cells per well in a 96-well plate (Corning, USA). Following incubation for 1, 2, 3, 4 or 5 days, the culture medium was discarded, and fresh CCK-8 working solution was prepared by diluting CCK-8 reagent (40203ES60, Yeasen, Shanghai, China) in complete culture medium at a ratio of 1:10. Then, 100 µl of the working solution was added to each well, and the plate was incubated at 37 °C in the dark for 1 h. Finally, the absorbance at 450 nm was measured by a SpectraMax iD5(Molecular Devices, CA, USA).

### 5‑Ethynyl‑2′‑deoxyuridine (EdU) assay

Pre-treated or transfected cells were seeded in a 6-well plate (Corning, USA) and cultured overnight. The cells were then treated with a Cell-Light EdU Apollo567 In Vitro Kit (Ribobio, Guangzhou, China), supplemented with 10 µM EdU reagent for 2 h. The cells were then fixed with 4% paraformaldehyde, permeabilized with 0.5% Triton X-100, and washed with PBS. Subsequently, the cells were incubated in the dark at room temperature with Alexa Fluor-488 for 30 min. After washing with PBS again, the cell nuclei were stained with DAPI. Finally, images were captured using an Olympus microscope (Tokyo, Japan).

### Wound healing assay

Pre-treated or transfected cells were seeded in a 6-well plate (Corning, USA) and cultured until they reached 80–90% confluency. The culture medium was then discarded, and the cells were gently washed with 100 µl of PBS to moisten the cell monolayer. Then, a scratch was made on the cell monolayer using a 200 µl pipette tip. The plate was washed with PBS buffer, and 2 mL of serum-free culture medium was added to each well before the plate was returned to the incubator for further culture. Finally, images of the wounds at 0 h and 24 h were captured using an Olympus microscope (Tokyo, Japan).

### Transwell assay

Cell migration and invasion assays were performed using Transwell chambers (8 μm pore size, Corning, USA). The upper chamber was either left uncoated for migration assays or coated with 100 µl of Matrigel (BD Biosciences, USA) for invasion assays. Approximately 100,000 pretreated or transfected cells were suspended in 100 µl of serum-free culture medium and seeded into the upper chamber of the Transwell insert, while 500 µl of complete culture medium was added to the lower chamber. After incubation for 24 h, the cells on the lower surface of the membrane were fixed with 4% paraformaldehyde and stained with 0.1% crystal violet (Vicmed, China).The cells on the upper surface of the membrane were removed using a cotton swab, and images were captured with a Leica Microsystems microscope (Mannheim, Germany) for cell counting.

### Colony forming assay

Approximately 200 pretreated or transfected cells were seeded into a 6-well plate (Corning, USA). After 14 days of incubation, the medium was aspirated, and the cells were fixed in 4% paraformaldehyde for 30 min. Subsequently, the cells were stained with 0.1% crystal violet and thoroughly washed with PBS. The plate was then placed on a light board for image capture, followed by colony counting.

### Hematoxylin-Eosin (HE) staining and immunohistochemistry (IHC)

Tissue samples were fixed at room temperature in 4% paraformaldehyde solution, dehydrated in ethanol solution, and embedded in paraffin. Tissue specimens were sectioned into 4 μm-thick slices, deparaffinized, stained with hematoxylin and eosin, and then dehydrated for mounting. Alternatively, after deparaffinization, antigen retrieval was performed, and nonspecific binding was blocked. Subsequently, primary antibodies were applied and incubated overnight at 4 °C, followed by incubation with the corresponding horseradish peroxidase-coupled anti-rabbit polymer (CST, USA) for 10 min at room temperature, and counterstaining with hematoxylin solution, Finally, representative images were captured and presented.

### RNA sequencing (RNA-seq)

To determine the gene expression profile of 786-O cells, total RNA was extracted and preserved using TRIzol (Invitrogen, USA). Subsequently, according to the manufacturer’s instructions, cDNA libraries were constructed using the TruSeq™ RNA Sample Prep Kit (Illumina, USA). Next, sequencing reads were aligned using HISAT2 software. Data analysis was performed using EdgeR software.

### Protein isolation of nuclear and cytoplasmic fractions

Cellular nuclear and cytoplasmic proteins were separated using a Nuclear and Cytoplasmic Protein Extraction Kit (P0028, Beyotime, Shanghai, China) according to the manufacturer’s instructions, and 786-O and A-498 cells were isolated from the nuclear and cytoplasmic fractions. The separated nuclear and cytoplasmic proteins were prepared for subsequent western blotting experiments, with GAPDH and Lamin B1 serving as internal controls.

### Western blotting

The obtained tissues or cells were lysed on ice for 30 min using lysis buffer (PC102, Epizyme, Shanghai, China). The protein concentration was determined using a BCA protein assay kit (ZJ101, Epizyme, Shanghai, China). Proteins (20 µg) were separated by SDS‒PAGE on 10% polyacrylamide gels and transferred onto NC membranes (WJ004, EpiZyme, Shanghai, China). The membranes were blocked with 5% skim milk at room temperature for 1 h, followed by overnight incubation with primary antibodies at 4 °C. After thorough washing, the membranes were incubated with secondary antibodies conjugated to horseradish peroxidase at room temperature for 1 h, and then subjected to membrane blotting using enhanced chemiluminescence detection reagents (NCM, Suzhou, China). Chemiluminescence signals were detected using an imaging system (AI600, GE, USA). The intensity of individual protein bands was measured using ImageJ software (NIH, Rasband, WS, USA). Detailed information about all antibodies used in this study is provided in Table S3.

### Coimmunoprecipitation (Co-IP) assay

An IP/Co-IP Kit (P2179S, Beyotime, Shanghai, China) was used to explore the physical interaction between SEC14L3 and RPS3. After cell lysis, the proteins were incubated with A/G magnetic beads prebound to the primary antibody at 4 °C overnight with gentle shaking. After incubation, the beads were separated using a magnetic rack, and the supernatant was discarded. The immunocomplexes were washed with prechilled lysis buffer (without protease inhibitors) to remove unbound proteins. The immunocomplexes were then eluted from the beads by heating at 100 °C for 10 min in 1× SDS loading buffer. Western blot analysis was performed on the eluted samples.

### GST pull-down assay

GST-SEC14L3 and His-RPS3 plasmids were transfected into *E. coli* separately to express the fusion proteins. Approximately 100 µg of GST or GST-SEC14L3 fusion proteins were immobilized in 50 µL of glutathione agarose and incubated together at 4 °C for 60 min. After three washes with PBST, around 100 µg of His-RPS3 fusion protein was added to the immobilized GST and GST-SEC14L3. The fusion proteins were gently rotated and incubated overnight at 4 °C. Bound proteins were eluted using an elution buffer (PBS containing 10 mM glutathione, pH 8.0) and analyzed by immunoblotting. The sequences of His-RPS3 and GST-SEC14L3 are listed in Table S4.

### In vivo ubiquitination assay

For endogenous ubiquitination, cell lysates from cells transfected with the designated plasmids were added to protein A/G agarose beads preadsorbed with an RPS3 antibody. Immunoprecipitation was performed, followed by the detection of ubiquitinated RPS3 protein through immunoblotting.

### Chromatin immunoprecipitation (ChIP) assay

The cells were treated with 1% formaldehyde at room temperature for 10 min to cross-link DNA. After sonication, the chromatin was precleared with A/G agarose beads to remove cross-linked chromatin. Subsequently, immunoprecipitation was carried out overnight at 4 °C using anti-NFKB1 antibody. IgG was used as a negative control, while RNA polymerase II was used as a positive control. Specific primers targeting the human SEC14L3 promoter and enhancer sequences were used for amplification (details provided in Table S1). PCR products were identified by agarose gel electrophoresis.

### Synthesis of siRNA-SEC14L3@PDA@MUC12 nanoparticles (SSPM-NPs, referred to as NPs)

siRNA-SEC14L3 (2 µmol) dissolved in RNase-free water was mixed with 100 µl of commercial liposomes (40802ES01, Yeasen, China) and vortexed for 30 s to form siRNA-SEC14L3@liposome (SS). The resulting suspension was poured into 10 mL of Tris-HCl (pH 8.8; 10 mM) solution, followed by the addition of dopamine hydrochloride (5 mg), to form polydopamine (PDA) (Adamas-beta, China) modified liposomes. The mixture was stirred at room temperature for 3 h to obtain PDA-modified liposomes (siRNA-SEC14L3@PDA, SSP). After centrifugation at 8,000 rpm for 10 min, the pellets were washed with distilled water. Subsequently, the obtained mixture was added to 1 ml of streptavidin solution (2 mg/mL) and shaken at 4 °C in the dark for 24 h to synthesize siRNA-SEC14L3@PDA@Streptavidin (SSPs). Finally, the obtained SSPs were mixed with 1 ml of biotinylated MUC12 antibody solution (50 µg/ml; Bioss, China) and shaken at 4 °C for 1 h. After centrifugation at 8,000 rpm for 10 min, the pellets were dispersed in 2 mL of distilled water and stored at 4 °C for further use.

### Characterization, particle size and zeta potential of NPs

The NPs were characterized using transmission electron microscopy (TEM) (HT7700, Hitachi, Japan) and energy-dispersive X-ray spectroscopy (EDS) elemental mapping.

For determining the particle size and zeta potential, 10 µl of SSP and NPs were dispersed in 1 mL of distilled water separately, and the samples were measured using a particle size analyzer (Nano ZS90, Worcestershire, UK) for zeta potential and particle size.

### Determination of MUC12 incorporation

Separately, 16 µl of SSP and NPs samples were mixed with 4 µl of 5× protein loading buffer and boiled at 100 °C for 10 min. Subsequently, the samples were loaded onto a 10% sodium dodecyl ulfate‒polyacrylamide gel for electrophoresis, with PDA serving as the negative control. After electrophoresis, the gel was stained with Coomassie Brilliant Blue staining solution until clear bands appeared, followed by rinsing with distilled water and gel imaging. The MUC12 band was observed at approximately 62 kDa.

### Cytophagy of the NPs

786-O cells were cultured in a 6-well plate until they reached 70–80% confluence. NPs were added to the cells in 2 mL of 1640 medium and cultured overnight. The following day, after washing with PBS, the cells were trypsinized and then collected by centrifugation in a 1.5 mL Eppendorf tube to allow the cell aggregates to settle at the bottom of the tube. The cell aggregates were fixed with 2.5% glutaraldehyde fixative and stored overnight at 4 °C. Subsequently, the cell aggregates were washed and dehydrated in Spurr’s low-viscosity resin at 60 °C for 2 days. Finally, ultrathin sections were cut, stained with lead citrate, and imaged using transmission electron microscopy.

### Animal models

Subcutaneous tumor and lung metastasis models were established in 5-week-old female BALB/c nude mice (Charles River, China). To establish a subcutaneous tumor model, 1,000,000 stable SEC14L3-knockdown A-498 cells and 1,000,000 A-498 cells transfected with negative control virus were separately injected into the left inguinal region of the mice. Tumor volumes were measured every 3 days using the formula: Volume (mm^3^) = 0.5 × width^2^ × length. At the endpoint, the weight of each tumor in all mice was recorded. For the lung metastasis model, 100,000 cells mentioned above were injected into the bloodstream via the tail vein. After 3 weeks, the mice were intraperitoneally injected with 100 mg/kg D-luciferin (Goldbio, USA), and images were captured using the AniView100 imaging system (Guangzhou, China). In the sunitinib treatment model, mice were intravenously injected with PBS, sunitinib, NPs, or NPs + sunitinib (10 nmol) every three days (200 µl). All animal experiments were conducted following protocols approved by the Animal Research Ethics Committee of the Tenth People’s Hospital of Shanghai.

### Statistical analysis

Statistical analysis was performed with GraphPad Prism 9.0 (La Jolla, CA, USA) and SPSS 13.0 (Chicago, IL, USA). Student’s t-test, analysis of variance (ANOVA), the chi-square test, and Kaplan‒Meier analysis were used for statistical comparisons. Each experiment was repeated at least three times. The data are presented as mean ± standard deviation (SD). A p-value < 0.05 was considered to indicate statistical significance.

## Results

### SEC14L3 is overexpressed in ccRCC and is a clinicopathological predictor

**Fig. 1:**
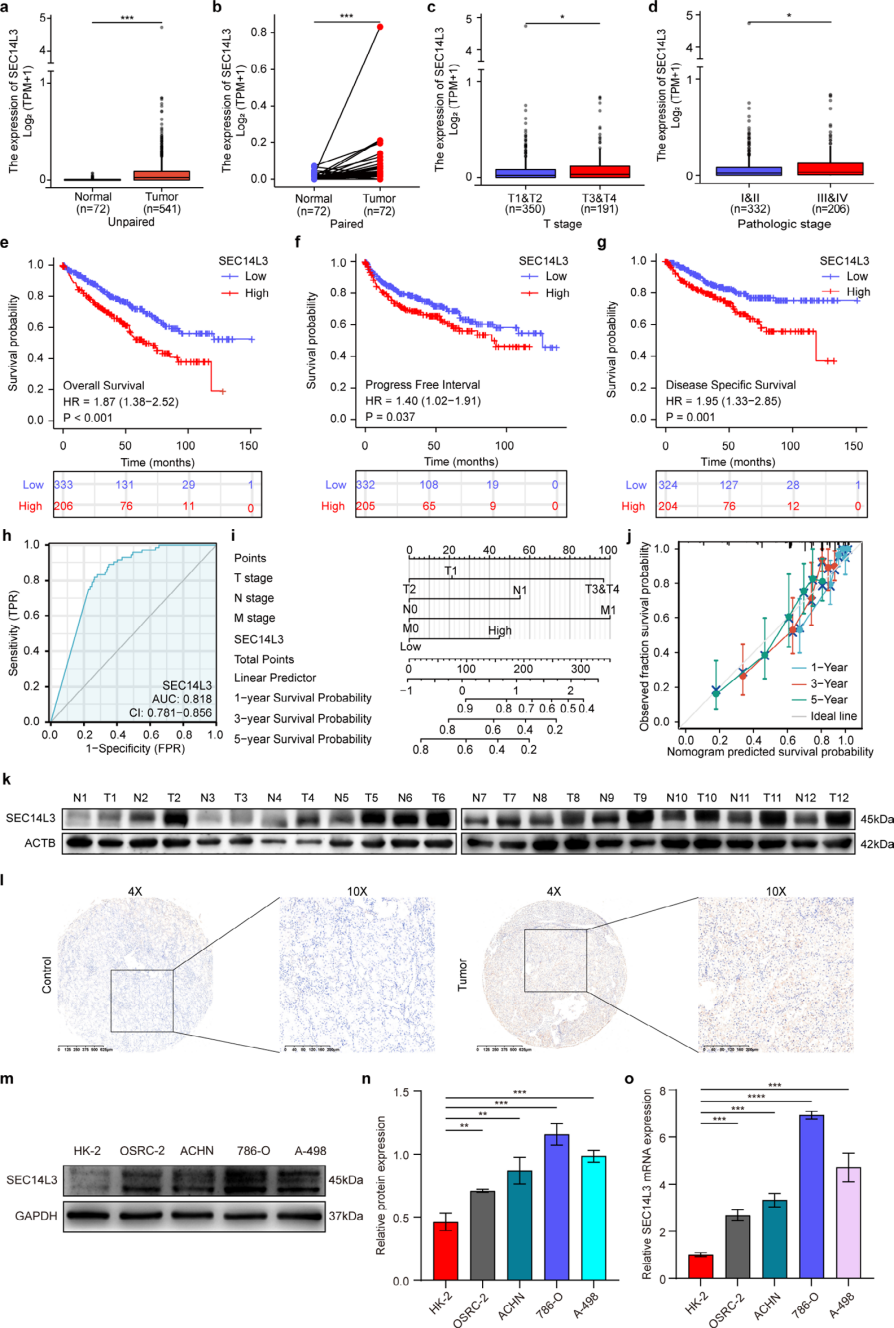
SEC14L3 is highly expressed in ccRCC and serves as an independent risk factor for the prognosis of ccRCC patients. **a**,** b.** TCGA cohort analyses of the SEC14L3 expression in ccRCC tumor samples and unpaired (**a**) or paired (**b**) normal tissues. **c**,** d.** The expression of SEC14L3 in different stages: tumor stage(**c**), pathological stage(**d**). **e-g.** Overall survival (**e**), Progress free interval (**f**) and disease specific survival (**g**) curve of ccRCC patients with low and high SEC14L3 expression. **h.** ROC curve of SEC14L3 between ccRCC and normal tissues. **i**,** j.** nomogram (**i**) and calibration curve analyses (**j**) of SEC14L3 in ccRCC patients. **k.** Western blot analysis of SEC14L3 expression levels in 12 ccRCC tissues and paired adjacent tissues. **l.** IHC staining image showing SEC14L3 expression in ccRCC. **m-o.** Western blot (**m**,** n**) and qRT-PCR (**o**) analyses of SEC14L3 expression levels in HK-2, OSRC-2, ACHN, 786-O, A-498 cells. *p < 0.05, **p < 0.01, ***p < 0.001, ****p < 0.0001

To explore the potential involvement of SEC14L3 in the clinical progression of ccRCC, we initially conducted a comprehensive analysis of SEC14L3 expression utilizing the TCGA database. Our findings revealed a notable upregulation of SEC14L3 expression in ccRCC tissues in both paired and unpaired samples (Fig. 1a, b). Then we analyzed SEC14L3 expression in GSE53757 dataset from GEO database and discovered that SEC14L3 was significantly upregulated in tumor tissues compared to adjacent non-tumor kidney tissues (Figure S1a). Next, we investigated the TCGA-ccRCC dataset to gain further insights into the dynamics of SEC14L3 expression across different stages of ccRCC progression. Intriguingly, our analysis revealed a significant increase in SEC14L3 expression in the T3&4 tumor grade group relative to that in the T1&2 group (Fig. 1c). Similarly, SEC14L3 expression exhibited a discernible increase in the pathological stage III&IV group compared to that in the I&II group (Fig. 1d). These findings collectively suggest a potential association between elevated SEC14L3 expression and clinical advancement in ccRCC patients.

Moreover, Kaplan‒Meier survival analysis of the TCGA cohort revealed a compelling association between elevated SEC14L3 expression in ccRCC patients and markedly shorter overall survival (OS), progression-free interval (PFI), and disease-specific survival (DSS) durations (Fig. 1e–g). Similarly, high expression of SEC14L3 was associated with reduced survival rates in GSE29609 dataset from GEO database (Figure S1b). Subsequent ROC analysis underscored the potential utility of SEC14L3 as a robust prognostic marker for predicting ccRCC progression (Fig. 1h). Furthermore, the nomogram (Fig. 1i), calibration curve analysis (Fig. 1j) and Cox regression analysis (Table 1) provided additional evidence confirming the independent prognostic significance of elevated SEC14L3 expression in ccRCC patients, solidifying its status as an independent risk factor for prognosis.

In summary, our analysis of the TCGA database revealed a significant correlation between elevated SEC14L3 expression and tumor grade and pathological stage in ccRCC patients. Importantly, our findings underscore the pivotal role of SEC14L3 as an independent prognostic indicator for ccRCC patients, highlighting its potential utility in the prognostic assessment of this malignancy.

**Table 1:** The results of Cox regression analysis

Characteristics	Total(*N*)	Univariate analysis			Multivariate analysis	
		Hazard ratio (95% CI)	*P* value		Hazard ratio (95% CI)	*P* value
T stage	539					
T1	278	Reference				
T2	71	1.517 (0.909–2.530)	0.111		0.799 (0.389–1.642)	0.542
T3&T4	190	3.594 (2.557–5.052)	**< 0.001**		2.215 (1.308–3.749)	**0.003**
N stage	257					
N0	241	Reference				
N1	16	3.453 (1.832–6.508)	**< 0.001**		1.786 (0.913–3.496)	0.090
M stage	506					
M0	428	Reference				
M1	78	4.389 (3.212–5.999)	**< 0.001**		2.865 (1.754–4.679)	**< 0.001**
SEC14L3	539					
Low	269	Reference				
High	270	1.498 (1.105–2.031)	**0.009**		1.603 (1.042–2.465)	**0.032**

### Knockdown of SEC14L3 suppresses the proliferation and metastasis of ccRCC both in vitro and in vivo

Prior to investigating the impact of SEC14L3 on the cellular phenotype of ccRCC, we assessed SEC14L3 protein expression levels in 12 pairs of clinically matched tumor and adjacent tissue samples (Fig. 1k). The results showed a significant increase in SEC14L3 expression in ccRCC tissues (Figure S1c). These findings were corroborated through IHC staining (Fig. 1l). Furthermore, we evaluated SEC14L3 protein and mRNA expression levels in normal renal tubular epithelial cells (HK-2) and four ccRCC cell lines (OSRC-2, ACHN, 786-O and A-498) (Fig. 1m-o). Our analyses revealed an analogous increase in SEC14L3 protein and mRNA levels in ccRCC cells, indicating a potential tumorigenic role for SEC14L3 in ccRCC.

**Fig. 2:**
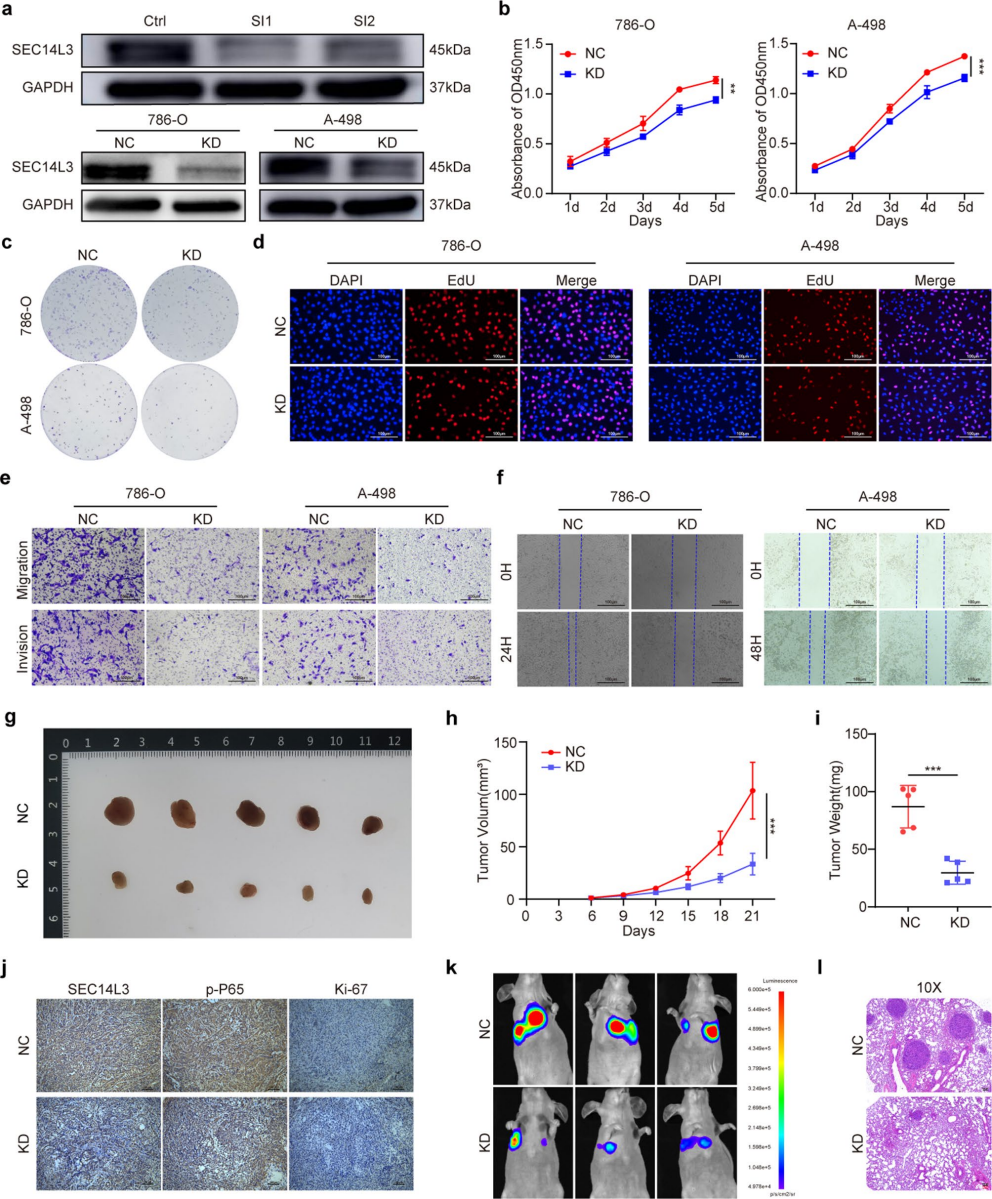
Knockdown of SEC14L3 suppresses the proliferation and metastasis of ccRCC both in vitro and in vivo. **a.** Western blot analysis for SEC14L3 expression in 786-O cells by siRNA (**a** top), or in 786-O and A-498 cells by lentiviral vectors (**a** bottom). **b-d.** CCK-8 assay (**b**), Colony formation assay (**c**) and EdU assay (**d**) were performed to evaluate the proliferation capacity of 786-O KD and A-498 KD cells. **e**,** f**. Transwell assay (**e**) and wound healing assay (**f**) were performed to evaluate cell migration and invasion capabilities of 786-O KD and A-498 KD cells. **g.** Image of xenograft tumors were taken 3 weeks after injection. **h**,** i.** Analyses of xenograft tumor volume (**h**) and weight (**i**). **j.** IHC was conducted to assess protein levels of Ki-67, SEC14L3, and p-P65 in xenograft tumors. **k.** Metastasis images were captured by an in vivo bioluminescence imaging system. **l.** H&E staining of lung metastatic tumors. Data are presented as mean ± SD of three independent experiments. **p < 0.01, ***p < 0.001

Given that SEC14L3 expression is relatively high in 786-O and A-498 cell lines, we selected these two cell lines for subsequent experiments. We knocked down SEC14L3 expression in 786-O cells through siRNA-SEC14L3-1 (SI1) and siRNA-SEC14L3-2 (SI2) transfection (Fig. 2a, S2a, b). Next, we synthesized lentiviral vectors harboring the most effective SI1 sequence to establish stable SEC14L3 knockdown 786-O and A-498 cells (Fig. 2a, S3a, b). Subsequently, we investigated the phenotypes of 786-O and A-498 cells transfected with si-SEC14L3 or SEC14L3 knockdown (KD) lentivirus. Cell proliferation was assessed via CCK-8 assays, colony formation assays, and EdU staining (Fig. 2b-d, S2c-g and S3c, d). Notably, diminished SEC14L3 expression resulted in slower growth, reduced colony formation, and a diminished proportion of EdU-positive cells in both the 786-O and A-498 cell lines. Furthermore, Transwell and wound healing assays were employed to evaluate cell migration and invasion capabilities (Fig. 2e, f, S2h-l and S3e-g). Remarkably, knockdown of SEC14L3 significantly attenuated the invasive and migratory capacities of 786-O and A-498 cells. Collectively, these findings provide compelling evidence implicating SEC14L3 in the inhibition of proliferation and metastasis in ccRCC cells in vitro.

To validate the impact of SEC14L3 on ccRCC cell proliferation in vivo, we subcutaneously injected SEC14L3 KD A-498 cells and corresponding negative control (NC) cells into BALB/c nude mice. Xenograft tumor models revealed discernible tumor growth inhibition in cells with diminished SEC14L3 expression, as evidenced by reduced tumor volumes and weights compared to those in the NC group (Fig. 2g-i). IHC staining revealed decreased levels of Ki-67 (a proliferation marker) and p-P65 (a downstream effector of SEC14L3) in the KD group (Fig. 2j). Subsequently, to explore the impact of SEC14L3 on metastasis in vivo, we established a lung metastasis model utilizing the aforementioned cells. Strikingly, SEC14L3 knockdown led to decreased lung bioluminescence intensity and fewer metastatic nodules (Fig. 2k, l). These results underscore the inhibitory role of SEC14L3 in ccRCC cell proliferation and metastasis in vivo. Hence, targeting SEC14L3 represents a promising strategy for impeding the progression of ccRCC, both in vitro and in vivo.

### Knockdown of SEC14L3 inhibits NFκB nuclear translocation and inactivates the NF-κB signaling pathway in ccRCC

**Fig. 3:**
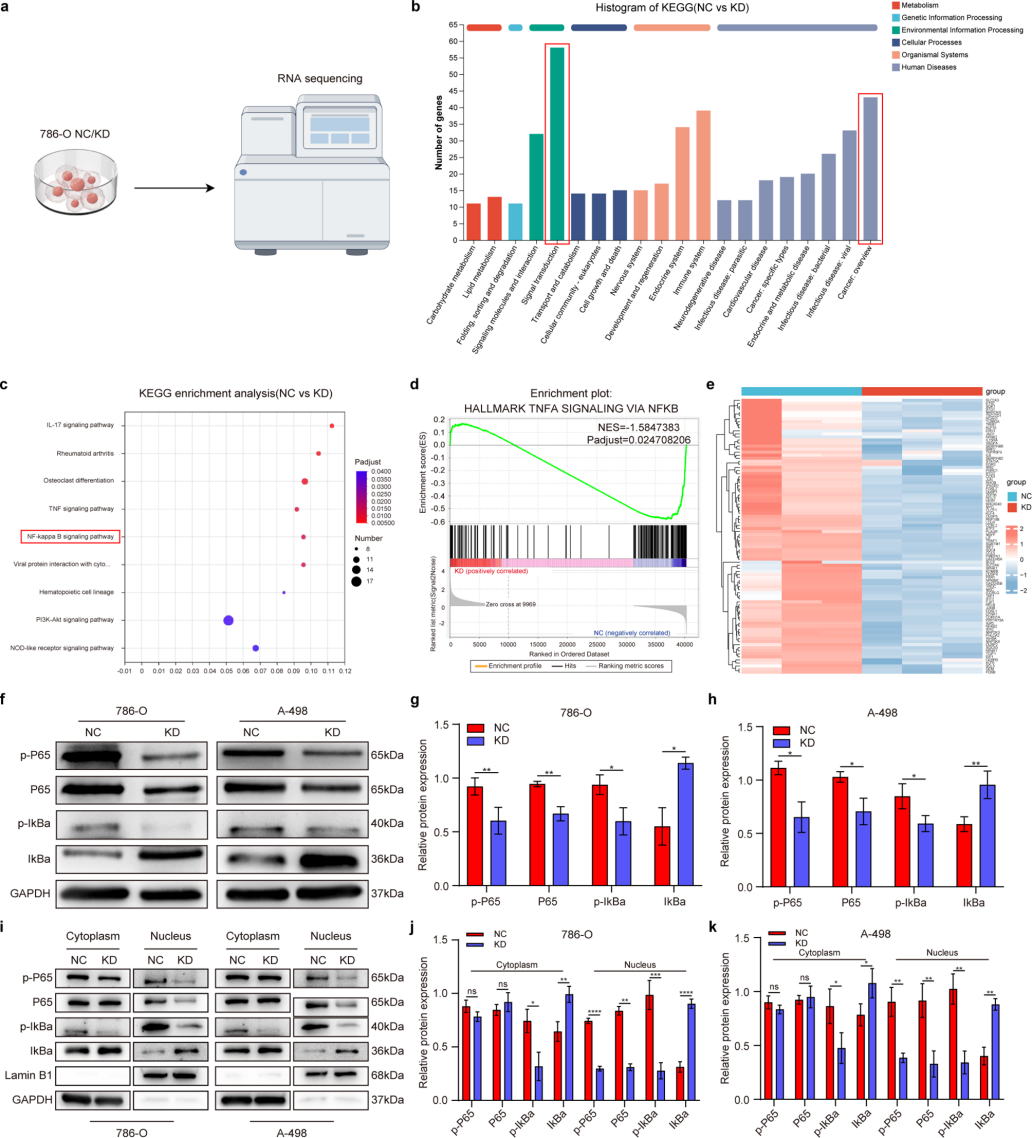
SEC14L3 knockdown inhibits NFκB nuclear translocation and inactivates the NF-κB signaling pathway in ccRCC. **a. **Schematic diagram of the RNA-seq experiment. **b**. KEGG annotations indicated that SEC14L3 was closely associated with cancer: overview and signal transduction. **c**. KEGG analysis revealed a close association between SEC14L3 and the NFκB signaling pathway. **d**. GSEA plot showed SEC14L3 KD level was negatively correlated with TNFα signaling via NFκB pathway. **e**. Heat maps of expression fold-change for the genes in TNFα signaling via NFκB pathway. Red signifies a higher fold-change, while blue signifies a lower fold-change. **f**-**h.** Western blot analysis of P65, p-P65, IκBα and p-IκBα expression levels in 786-O and A-498 KD cells. **i**-**k.** Western blot analyses of P65, p-P65, IκBα and p-IκBα expression levels in the cytoplasm and nucleus of 786-O and A-498 KD cells. Data are presented as mean ± SD of three independent experiments. NS not significant, *p < 0.05, **p < 0.01, ***p < 0.001, ****p < 0.0001

To elucidate the specific molecular mechanisms underlying SEC14L3-mediated modulation of ccRCC progression, we conducted RNA-seq analysis on SEC14L3 NC and KD 786-O cells (Fig. 3a). KEGG annotations revealed that SEC14L3 knockdown leads to increased alterations in cancer and is closely associated with signal transduction (Fig. 3b). Subsequent KEGG enrichment analysis revealed that genes altered following SEC14L3 knockdown were enriched in the NFκB signaling pathway(Fig. 3c, Fig. S4a). Gene set enrichment analysis (GSEA) further confirmed that the genes associated with the NFκB signaling pathway were downregulated in the SEC14L3 KD group (Fig. 3d, e). Collectively, these findings suggest that SEC14L3 may participate in ccRCC progression by modulating NFκB signaling pathway activation.

Furthermore, we assessed the expression of NFκB markers at the overall protein level in 786-O and A-498 cells (Fig. 3f-h). Compared to the NC group, the KD group exhibited diminished expression levels of P65, p-P65, and p-IκBα, accompanied by a significant increase in IκBα expression, indicating significant inhibition of the NFκB signaling pathway following SEC14L3 knockdown. Additionally, the precursor of P50, NFKB1, was also downregulated in the KD group (Figure S4b, c). Subsequently, we investigated the expression of these proteins in the cytoplasm and nucleus, revealing NFκB translocation to the cell nucleus (Fig. 3i-k). Intriguingly, compared to those in the NC group, no significant alterations in the expression of P65 or p-P65 in the cytoplasm were observed; however, their expression levels in the nucleus were markedly reduced. Moreover, the expression levels of p-IκBα were attenuated, while IκBα expression was increased, both in the cytoplasm and nucleus.

In summary, our findings indicate that knocking down SEC14L3 in ccRCC cells reduces the phosphorylation of IκBα, enhancing its accumulation and interaction with the P50/P65 heterodimer. This interaction hinders the heterodimer’s nuclear localization signal, inhibiting its translocation. Consequently, there is reduced nuclear P65 accumulation and attenuated DNA binding activity, leading to NFκB signaling pathway inhibition.

### SEC14L3 interacts with RPS3 and negatively regulates its protein level through ubiquitination

**Fig. 4:**
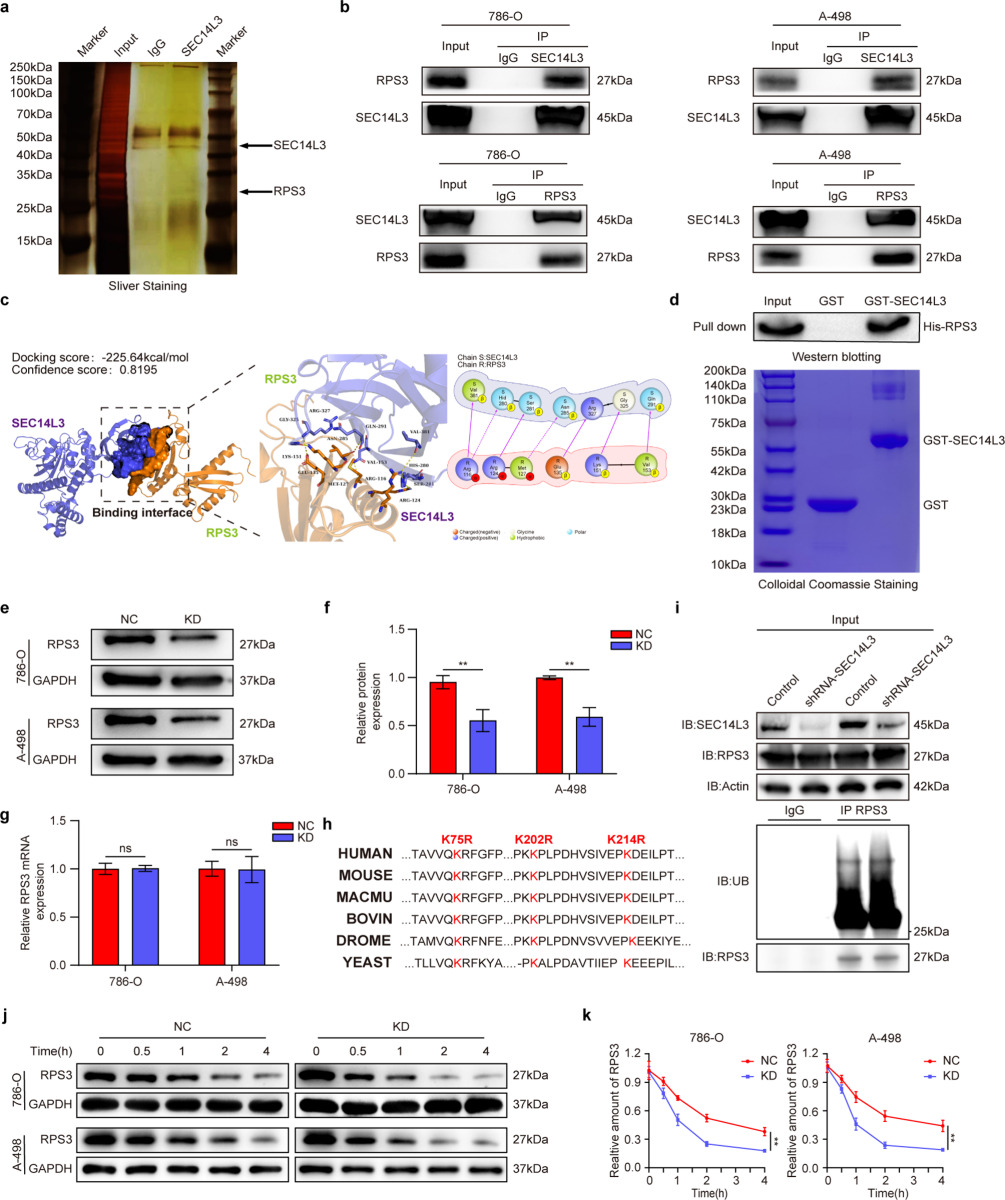
SEC14L3 interacts with RPS3 and negatively regulates its protein level through ubiquitination. **a**. Silver staining of SEC14L3 immunoprecipitation lysates. Arrows denote distinct bands observed in immunoprecipitation assays between the SEC14L3 and IgG groups. **b**. 786-O and A-498 cells were exposed to MG132 (10µM) for 8 h and subsequently harvested. Cell lysates underwent co-immunoprecipitation followed by western blot analysis. **c.** PyMOL software illustrates the interaction between SEC14L3 and RPS3 within their respective 3D protein structures. **d.** Detection of His-RPS3 bound to GST-SEC14L3 or GST in a GST pull-down assay. (top). The expression of GST and GST-SEC14L3 were verified through Colloidal Coomassie staining (bottom). **e-g.** Western blot (**e**,** f**) and qRT-PCR (**g**) analyses of RPS3 expression levels in 786-O, A-498 KD cells. **h.** Alignment of amino acid sequences for K75, K202, and K214 in RPS3 across various species. **i.** HEK-293T cells were transfected with sh-SEC14L3 plasmids for 24 h, then treated with MG132 (10µM) for 8 h before harvesting. Cell lysates underwent co-immunoprecipitation using an anti-RPS3 antibody, followed by western blot analysis. **j**,** k.** NC and KD cells were collected for western blot analysis after being treated with 100 µg/mL cycloheximide (CHX) for 0.5, 1, 2, and 4 h (**j**). The intensity of RPS3 bands was measured by ImageJ software (**k**). Data are given as the means ± SD of three independent experiments. NS not significant, **P < 0.01

To elucidate the molecular mechanism underlying SEC14L3-mediated inhibition of the NFκB signaling pathway, we utilized immunoprecipitation coupled with shotgun proteomic analysis to identify SEC14L3-interacting proteins. Silver staining of the immunoprecipitated samples revealed several discernible protein bands in the SEC14L3 group compared to the IgG control group (Fig. 4a). Among these bands, the specific expression of RPS3 was particularly pronounced. Previous studies have highlighted RPS3’s crucial role as a non-Rel subunit of NFκB, promoting its interaction with the NFκB p65 subunit in the nucleus, thereby stabilizing the activated state of NFκB [23, 24]. Furthermore, RPS3 has been implicated in NFκB pathway activation by mediating IκBα ubiquitination [31]. Therefore, our subsequent investigations aimed to elucidate the role of RPS3 in SEC14L3-mediated the regulation of the NFκB signaling pathway.

Following the identification of RPS3 as a potential interacting partner of SEC14L3, we conducted coimmunoprecipitation (co-IP) experiments to validate the physical interaction between these proteins. The results unequivocally confirmed the endogenous binding of SEC14L3 with RPS3 (Fig. 4b). Subsequently, we performed protein-protein docking analysis to elucidate the structural basis of the SEC14L3-RPS3 interaction (Fig. 4c). Next, to investigate whether SEC14L3 directly binds to RPS3, we performed a GST pull-down assay in an E. coli system using GST-SEC14L3 and HIS-RPS3. Purified proteins were confirmed by SDS-PAGE (Figures S5a, b). The results demonstrated that RPS3 directly binds to GST-SEC14L3, but not to GST alone (Fig. 4d). Collectively, these findings underscore a robust association between the SEC14L3 and RPS3 proteins at the molecular level.

Subsequently, we validated the expression of RPS3 in SEC14L3 KD 786-O and A-498 cells. Remarkably, the results demonstrated a significant decrease in RPS3 expression at the protein level following SEC14L3 knockdown (Fig. 4e, f), while no significant change was observed in RPS3 expression at the mRNA level (Fig. 4g). This finding implies that SEC14L3 is involved in regulating the stability of RPS3 through posttranscriptional mechanisms. Ubiquitin-modified lysine residues remain consistent throughout eukaryotes, with multiple particular lysine sites identified as monoubiquitination sites on human RPS3 [32], and residues K75R, K202R, and K214R are potential ubiquitination sites on RPS3 ((Fig. 4h). Therefore, we sought to explore whether SEC14L3 modulates the stability of RPS3 via ubiquitination. Initially, we investigated alterations in endogenous ubiquitination. Encouragingly, the results revealed an increase in the endogenous ubiquitination level of RPS3 following SEC14L3 knockdown (Fig. 4i). Additionally, cycloheximide (CHX) was added to both the NC and KD group to inhibit protein synthesis in 786-O and A-498 cells. Consistent with these findings, SEC14L3 KD led to a shortened half-life of endogenous RPS3 compared to the NC group (Fig. 4j, k). In subsequent rescue experiments, we found that MG132, instead of Chloroquine (CQ), eliminated the down-regulation of RPS3 expression in KD group (Figure S5c-f). In conclusion, our results substantiate the notion that SEC14L3 interacts with RPS3, thereby regulating its ubiquitination and subsequent degradation. Consequently, SEC14L3 could exert its regulatory influence on NFκB activation by modulating the ubiquitination of RPS3.

### Inactivation of the NFκB signaling pathway can reciprocally suppress the expression of SEC14L3

**Fig. 5:**
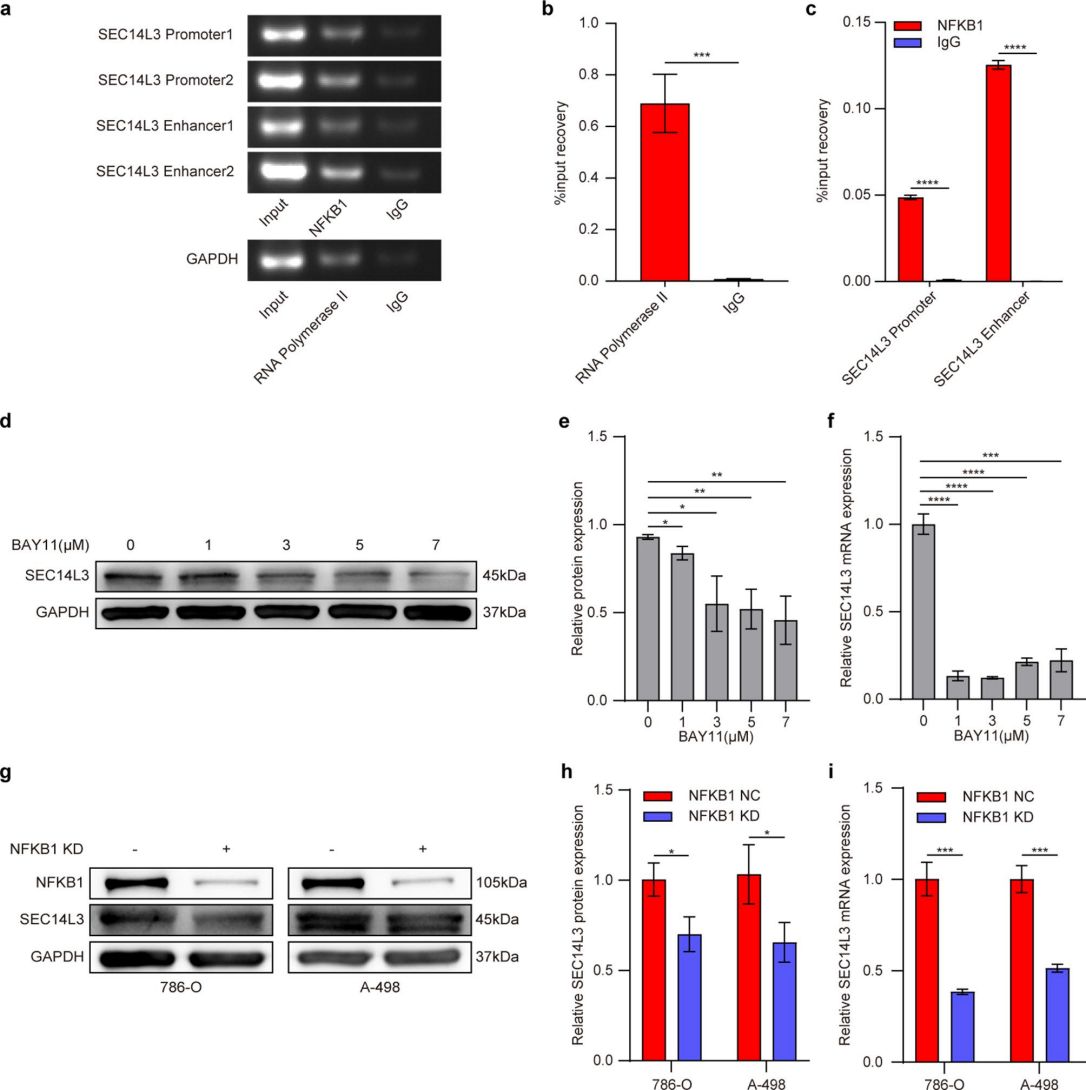
Inactivation of the NFκB signaling pathway can reciprocally suppress the expression of SEC14L3. **a.** ChIP assays reveal the binding of NFKB1 to potential binding sites within the SEC14L3 promoter and enhancer regions. **b**,** c.** qRT-PCR analysis of RNA polymerase II (positive control) and IgG (negative control) (**b**) along with SEC14L3 promoter and enhancer (**c**) in ChIP assays. **d-f.** 786-O cells were treated with BAY 11 in different concentrations for 24 h, followed by Western blot (**d**,** e**) and qRT-PCR (**f**) analyses. **g-i.** 786-O and A-498 cells were infected with NFKB1 knockdown lentiviral vectors for 78 h. Cells were collected for Western blot (**g**,** h**) and qRT-PCR (**i**) analyses. Data are presented as mean ± SD of three independent experiments. NS not significant, *p < 0.05, **p < 0.01, ***p < 0.001, ****p < 0.0001

Due to the role of NFκB as a protein complex that controls DNA transcription [33, 34] and its involvement in the expression of various proteins, we hypothesized that inactivation of the NFκB signaling pathway might influence the transcription of SEC14L3 and subsequent protein expression (https://genome.ucsc.edu/). To validate this hypothesis, we identified four putative NFKB1 response elements (SEC14L3 Promoter1, 2, and Enhancer1, 2). ChIP experiments demonstrated that NFKB1 can bind to these putative response elements (Fig. 5a-c), indicating that NFKB1 regulates SEC14L3 expression by binding to its promoter and enhancer regions.

To corroborate the aforementioned findings, we treated 786-O cells with varying concentrations of the NFκB signaling pathway inhibitor BAY11. The results revealed a dose-dependent reduction in SEC14L3 protein expression with increasing concentrations of BAY11 (Fig. 5d, e), concomitant with a significant decrease in SEC14L3 mRNA levels following BAY11 treatment (Fig. 5f). Subsequently, we stably knocked down NFKB1 expression in 786-O and A-498 cells via lentiviral vectors (Fig. 5g). Notably, upon NFKB1 knockdown, both cell lines exhibited a substantial decrease in SEC14L3 expression at both the protein and mRNA levels (Fig. 5g-i). These findings collectively underscore the pivotal role of NFKB1 as a transcription factor in modulating SEC14L3 expression, whereby its inactivation results in significant suppression of SEC14L3 expression.

### Knockdown of SEC14L3 enhances the sensitivity of ccRCC cells to sunitinib treatment

**Fig. 6:**
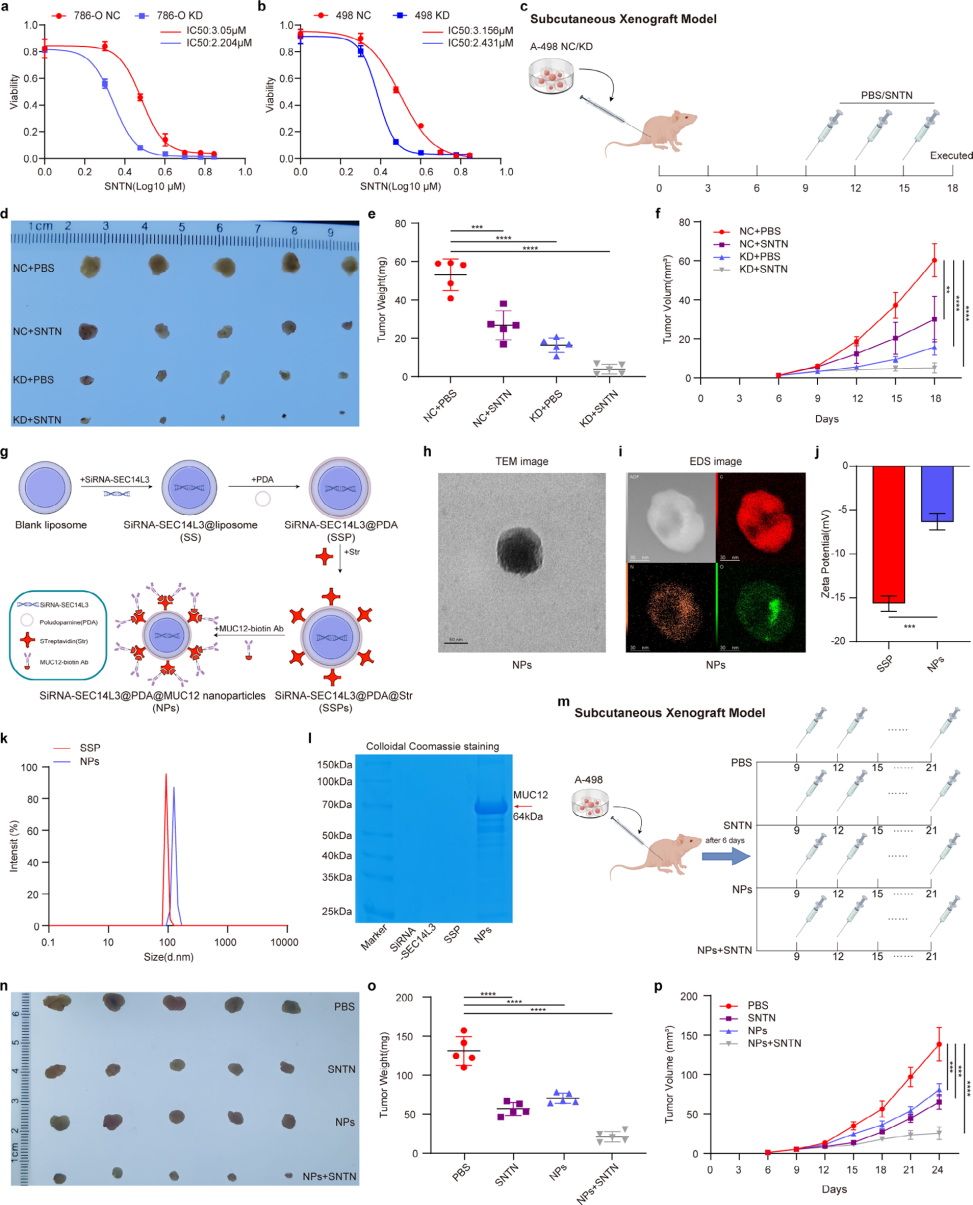
Knockdown of SEC14L3 enhances the sensitivity of ccRCC cells to sunitinib treatment. **a**,** b.** 786-O (**a**) and A-498 (**b**) NC&KD cells were treated with different concentrations of sunitinib for 24 h. CCK-8 assay was utilized to evaluate the impact of SEC14L3 downregulation on the cytotoxicity induced by sunitinib. **c.** Schematic model of subcutaneous xenograft model with intravenous injection of PBS and sunitinib. **d.** Image of xenograft tumors were taken 18 days after injection. **e**,** f.** Analyses of xenograft tumor weight (**e**) and volume (**f**). **g.** Approaches to synthesize MUC12-targeted NPs. **h**,** i.** TEM (**h**) and EDS (**i**) imaging of NPs. **j**,** k.** Zeta potentials (**j**) and elevated diameters (**k**) of SSP and NPs. **l.** Colloidal Coomassie images of SSP and NPs. **m.** Schematic model of subcutaneous xenograft model with intravenous injection of PBS, sunitinib, NPs and sunitinib + NPs. **n.** Image of xenograft tumors were taken 3 weeks after injection. **o**,** p.** Analyses of xenograft tumor weight (**o**) and volume (**p**). **p < 0.01, ***p < 0.001, ****p < 0.0001

NFκB pathway activation contributes to chemoresistance in various tumors [35–37], including sunitinib resistance in RCC [29, 30]. Since sunitinib is the standard first-line therapy for RCC [30], exploring strategies to enhance its efficacy is crucial. Given the therapeutic effects of SEC14L3 knockdown on ccRCC and its inhibition of the NFκB pathway, we investigated whether combining SEC14L3 downregulation with sunitinib treatment could improve outcomes. We first evaluated whether SEC14L3 downregulation could increase ccRCC cell sensitivity to sunitinib. Drug sensitivity assays revealed reduced IC50 values of sunitinib in SEC14L3 KD 786-O and A-498 cells (Fig. 6a, b), indicating enhanced sensitivity to sunitinib with SEC14L3 knockdown. Xenograft models using A-498 NC and KD cells treated with a combination of sunitinib revealed a synergistic effect of SEC14L3 knockdown on sunitinib efficacy. Tumors treated with this combination exhibited the smallest volume and weight (Fig. 6c-f), demonstrating the superior therapeutic efficacy of this regimen.

Subsequently, we attempted to construct therapeutic nanocomplexes encapsulating siRNA-SEC14L3 within lipid nanoparticles externally bound to the targeted antibody MUC12 [38] for treating ccRCC, as illustrated in Fig. 6g. TEM imaging and EDS mapping demonstrated that, after successful PDA modification, the synthesized NPs exhibited nearly spherical shapes (Fig. 6h, i). Furthermore, due to the external attachment of MUC12, the surface zeta potential of the NPs decreased while the diameter increased, indicating improved stability, as an average diameter of approximately 120 nm (Fig. 6j, k). Colloidal Coomassie blue staining revealed the presence of MUC12 on the surface of the NPs (Fig. 6l). Cytophagy experiments demonstrated successful internalization of the NPs into 786-O cells (Figure S6a), leading to effective downregulation of SEC14L3 and inhibition of malignant cell phenotypes in 786-O cells (Figure S6b-n). Subsequently, we evaluated the in vivo biocompatibility of the NPs in normal mice. There were showed no significant differences in blood urea nitrogen (BUN), creatinine, alanine transaminase (ALT) or aspartate transaminase (AST) levels between NPs group and the NC group (Figure S7a), and there were no apparent changes in organ morphology (Figure S7b).

After confirming the efficacy and biosafety of the NPs, we combined in vivo therapy utilizing NPs with sunitinib (Fig. 6m). In xenograft models, our results showed that, compared to the PBS group, both the sunitinib group and the NPs group exhibited decreased tumor weight and volume. However, the combination of NPs and sunitinib resulted in a remarkable reduction in tumor weight and volume (Fig. 6n-p). Thus, we successfully demonstrated that SEC14L3 could serve as a promising therapeutic target for combined treatment with sunitinib in ccRCC.

## Discussion

RCC is a prevalent solid tumor among adults, ranking among the top ten malignancies, with an incidence of 5% in men and 3% in women according to the latest cancer statistics [39]. ccRCC is the most common subtype of RCC and poses a significant threat to human health. However, the mechanisms underlying its initiation and progression remain largely elusive. Emerging evidence suggests that SEC14L3 may play a pivotal role in the development and progression of human cancers [16, 17]. However, the precise biological functions and molecular mechanisms of SEC14L3 in ccRCC remain poorly understood.

In this study, SEC14L3 was identified as a promising prognostic biomarker in clinical specimens of patients with ccRCC. Our analysis revealed a significant increase in SEC14L3 expression in ccRCC tissues compared to adjacent normal tissues. Further clinicopathological analysis revealed a correlation between high SEC14L3 expression and advanced tumor grade and pathological stage. Moreover, Kaplan‒Meier survival curves demonstrated poorer OS, PFI, and DSS in ccRCC patients exhibiting higher SEC14L3 expression levels. The area under the ROC curve (AUC) for SEC14L3 expression was 0.818, indicating its high accuracy in distinguishing ccRCC from normal tissues. Collectively, these findings underscore the potential of SEC14L3 as an effective tumor biomarker in ccRCC, and offer valuable insights for prognostic assessment and clinical management.

Aberrant activation of the NFκB signaling pathway has been implicated in processes such as cell proliferation and metastasis [40–42]. Previous studies have implicated phosphatidylinositol transfer proteins in the regulation of the NFκB signaling pathway [43]. However, there is a paucity of research regarding the involvement of SEC14L3 in the activation of the NFκB signaling pathway. In this study, our findings revealed that the downregulation of SEC14L3 augmented the accumulation of IκBα in cells, consequently inhibiting the nuclear translocation of P65, thereby transcriptionally inactivating downstream target genes of the NFκB signaling pathway and inhibiting the progression and metastasis of ccRCC. However, upon inactivation of NFκB, the expression of NFKB1 was suppressed, consequently leading to a further reduction in SEC14L3 expression. These findings shed light on the intricate regulatory interplay between SEC14L3 and the NFκB signaling pathway in the context of ccRCC progression and metastasis.

In recent years, accumulating evidence has underscored the regulatory role of RPS3 in NFκB activity [44–46]. RPS3 interacts with IκBα in resting cells and maintains the RPS3 pool in the NFκB signaling pathway [46]. Notably, in colorectal cancer, the RPS3-IκBα interaction modulates IκBα ubiquitination, thereby influencing NFκB pathway activation [31]. Moreover, RPS3 itself acts as a substrate for ubiquitination, contributing to NFκB regulation. For instance, circPLCE1-411 facilitates the ubiquitination and degradation of RPS3 through the HSP90α/RPS3 complex, resulting in NFκB pathway inactivation [47]. Additionally, the E2-E3 complex composed of UBE2J1/TRIM25 targets RPS3 for ubiquitination and degradation at the K214 residue, further leading to NFκB pathway inactivation [48]. In our study, we identified SEC14L3 as an upstream protein that interacts with RPS3. Knocking down SEC14L3 facilitated the ubiquitination and subsequent degradation of RPS3 through posttranscriptional mechanisms, ultimately resulting in NFκB pathway inactivation. Thus, our study revealed a novel SEC14L3-RPS3-NFκB loop, in which the downregulation of SEC14L3 expression activates this loop, suppressing ccRCC proliferation and metastasis. However, further investigation is needed to elucidate the specific residues involved in SEC14L3-induced ubiquitination and degradation of RPS3, as well as the interaction of RPS3 with IκBα in ccRCC.

Sunitinib is a molecular targeted therapeutic drug used as a first-line chemotherapy for metastatic RCC [49]. However, despite initial response rates of up to 47% to sunitinib [50, 51], resistance and tumor progression frequently develop after 9 to 12 months of treatment [52, 53]. Our study revealed that knockdown of SEC14L3 significantly enhances the sensitivity of ccRCC cells to sunitinib, with the most pronounced therapeutic effect observed when SEC14L3 knockdown is combined with sunitinib treatment. The emergence of nanotechnology has provided possibilities for gene therapy in tumor diseases [54–56]. Liposomes and PDA nanoparticles are considered ideal carriers for gene transfer therapy due to their excellent biocompatibility and biodegradability [57, 58]. In our study, we utilized liposomes and PDA nanoparticles encapsulating SEC14L3 siRNA for the treatment of ccRCC. Our results demonstrated that the NPs effectively knocked down SEC14L3 expression in ccRCC and further inhibited the growth of subcutaneous tumors in mice when combined with sunitinib treatment. This novel treatment modality holds promise as a new approach for ccRCC therapy, potentially overcoming resistance to sunitinib and improving patient outcomes.

**Fig. 7:**
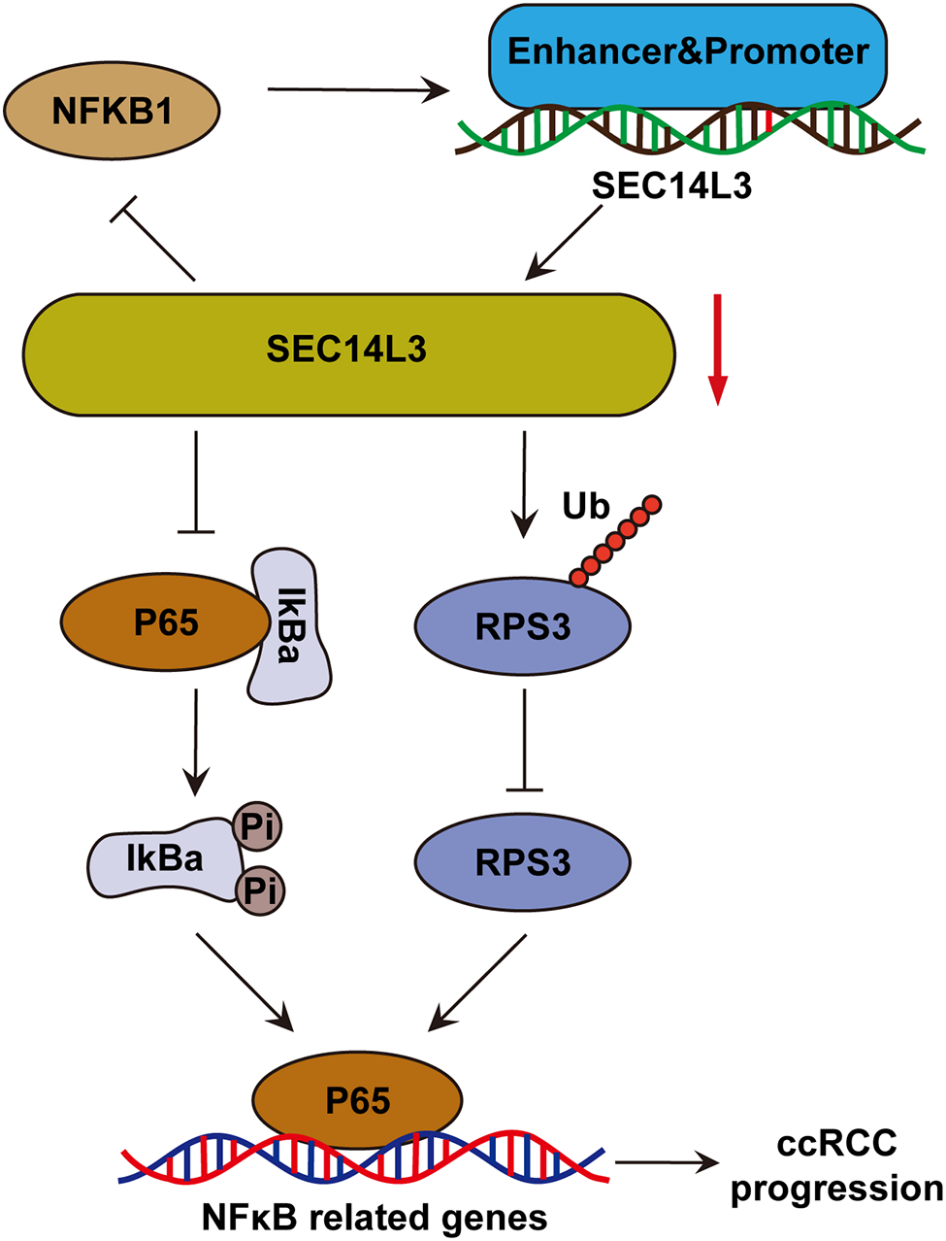
A schematic model delineating the mechanisms of SEC14L3 in ccRCC. In summary, knocking down SEC14L3 facilitated the ubiquitination-mediated degradation of RPS3, and augmented IκBα accumulation in ccRCC. These events collectively impede the nuclear translocation of P65, thereby inhibiting the activation of the NFκB signaling pathway and consequently restraining the proliferation and metastasis of ccRCC cells. Furthermore, diminished SEC14L3 levels exerted a suppressive effect on NFKB1 expression within the NFκB signaling cascade. the inhibited NFKB1, acting as a transcription factor in the SEC14L3 promoter and enhancer regions, further suppresses SEC14L3 expression, establishing a positive feedback loop of SEC14L3/RPS3/NFκB that collectively exerts inhibitory effects on ccRCC progression

## Conclusions

In summary, our research revealed that SEC14L3 is markedly upregulated in ccRCC and is correlated with poor prognosis in ccRCC patients. Mechanistically (Fig. 7), knocking down SEC14L3 facilitated the ubiquitination-mediated degradation of RPS3, and augmented IκBα accumulation in ccRCC cells. These events collectively impede the nuclear translocation of P65, thereby inhibiting the activation of the NFκB signaling pathway and consequently restraining the proliferation and metastasis of ccRCC cells. Furthermore, diminished SEC14L3 levels exerted a suppressive effect on NFKB1 expression within the NFκB signaling cascade. The inhibition of NFKB1, which acts as a transcription factor in the SEC14L3 promoter and enhancer regions, further suppresses SEC14L3 expression, establishing a positive feedback loop of SEC14L3/RPS3/NFκB that collectively inhibits ccRCC progression.

## Electronic supplementary material

Below is the link to the electronic supplementary material.

Supplementary Material 1

Supplementary Material 2

Supplementary Material 3

Supplementary Material 4

Supplementary Material 5

Supplementary Material 6

Supplementary Material 7

Supplementary Material 8

Supplementary Material 9

Supplementary Material 10

Supplementary Material 11

## Data Availability

The datasets used and/or analyzed during the current study are available from the corresponding authors (lm1191@126.com; wangkeyi0910@163.com; pengbo6908@163.com) upon reasonable request.

## Abbreviations

ccRCC: clear cell Renal Cell Carcinoma
RCC: Renal Cell Carcinoma
SEC14L3: SEC14-like 3
RPS3: Ribosomal Protein S3
SSPM-NPs (NPs): siRNA-SEC14L3@PDA@MUC12 nanoparticles
SSP: siRNA-SEC14L3@PDA
SSPs: siRNA-SEC14L3@PDA@Streptavidin
PDA: Polydopamine
MUC12: Mucin 12
NFκB: Nuclear Factor kappa B
GOLD: Golgi Dynamics
qRT‒PCR: Quantitative Real-Time PCR
siRNA: Small Interfering RNA
CCK-8: Cell Counting Kit-8
EdU: 5‑Ethynyl‑2′‑deoxyuridine
HE: Hematoxylin-Eosin
IHC: Immunohistochemistry
Co-IP: Coimmunoprecipitation
RNA-seq: RNA sequencing
ChIP: Chromatin Immunoprecipitation
TEM: Transmission Electron Microscopy
EDS: Energy-Dispersive X-ray Spectroscopy
TCGA: The Cancer Genome Atlas
GEO: Gene Expression Omnibus
KEGG: Kyoto Encyclopedia of Genes and Genomes
OS: Overall Survival
PFI: Progression-Free Interval
DSS: Disease-Specific Survival
SI1: siRNA-SEC14L3-1
SI2: siRNA-SEC14L3-2
NC: Negative Control
KD: Knockdown
GSEA: Gene Set Enrichment Analysis
SDS-PAGE: Sodium Dodecyl Sulfate–Polyacrylamide Gel
CHX: Cycloheximide
CQ: Chloroquine
BUN: Blood Urea Nitrogen
ALT: Alanine Aminotransferase
AST: Aspartate Aminotransferase
ROC: Rate of Change
AUC: Area Under the Curve
ANOVA: Analysis of Variance
SD: Standard Deviation
