# Supplementary figures and images for: SEC14L3 knockdown inhibited clear cell renal cell carcinoma proliferation, metastasis and sunitinib resistance through an SEC14L3/RPS3/NFκB positive feedback loop
Source: J Exp Clin Cancer Res. 2024 Oct 19;43:288. doi: 10.1186/s13046-024-03206-5 (PMC11490128; doi:10.1186/s13046-024-03206-5)

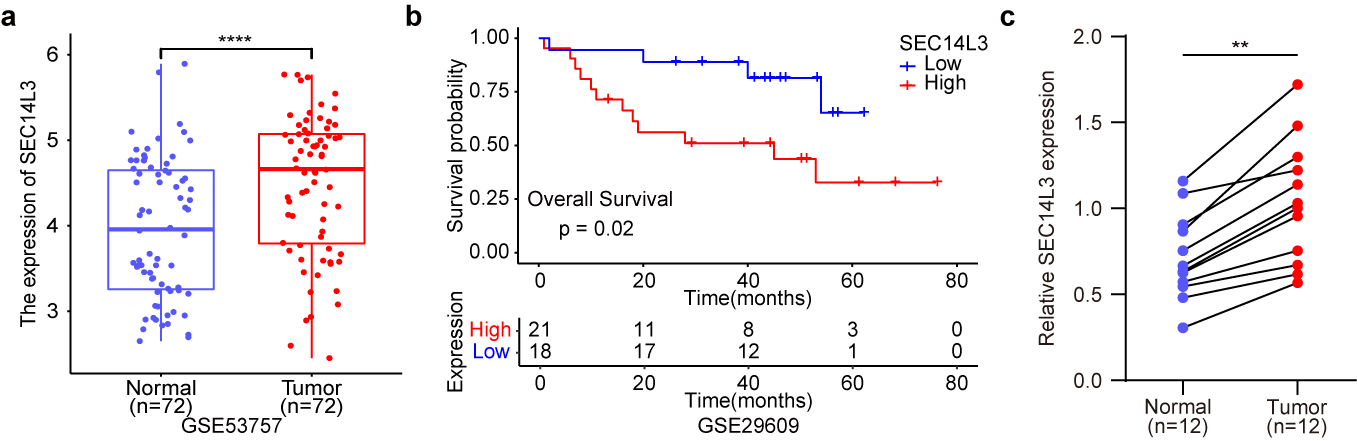

Supplement: Supplementary file 1 — Supplementary Material 1 [file 13046_2024_3206_MOESM1_ESM.tif]

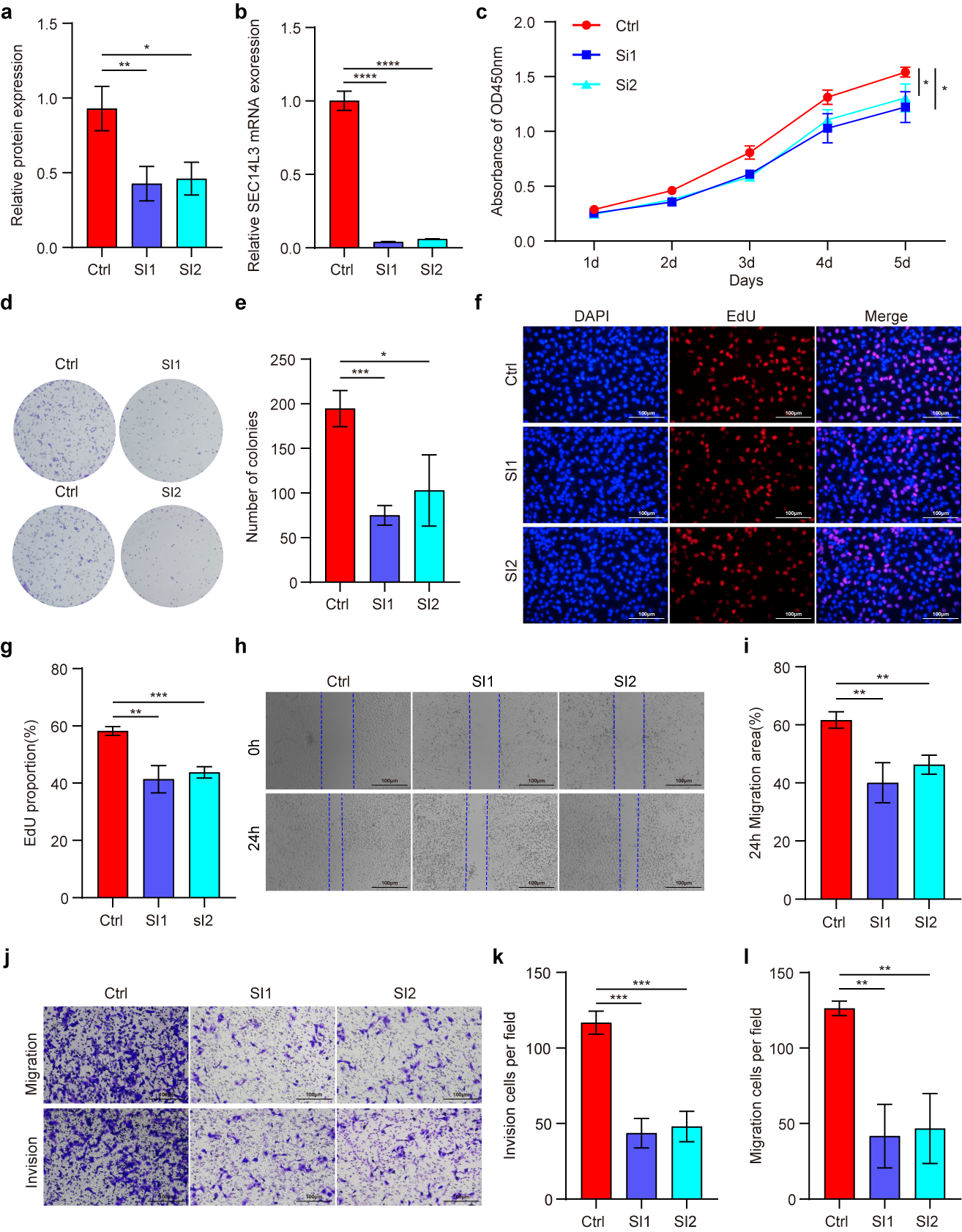

Supplement: Supplementary file 2 — Supplementary Material 2 [file 13046_2024_3206_MOESM2_ESM.tif]

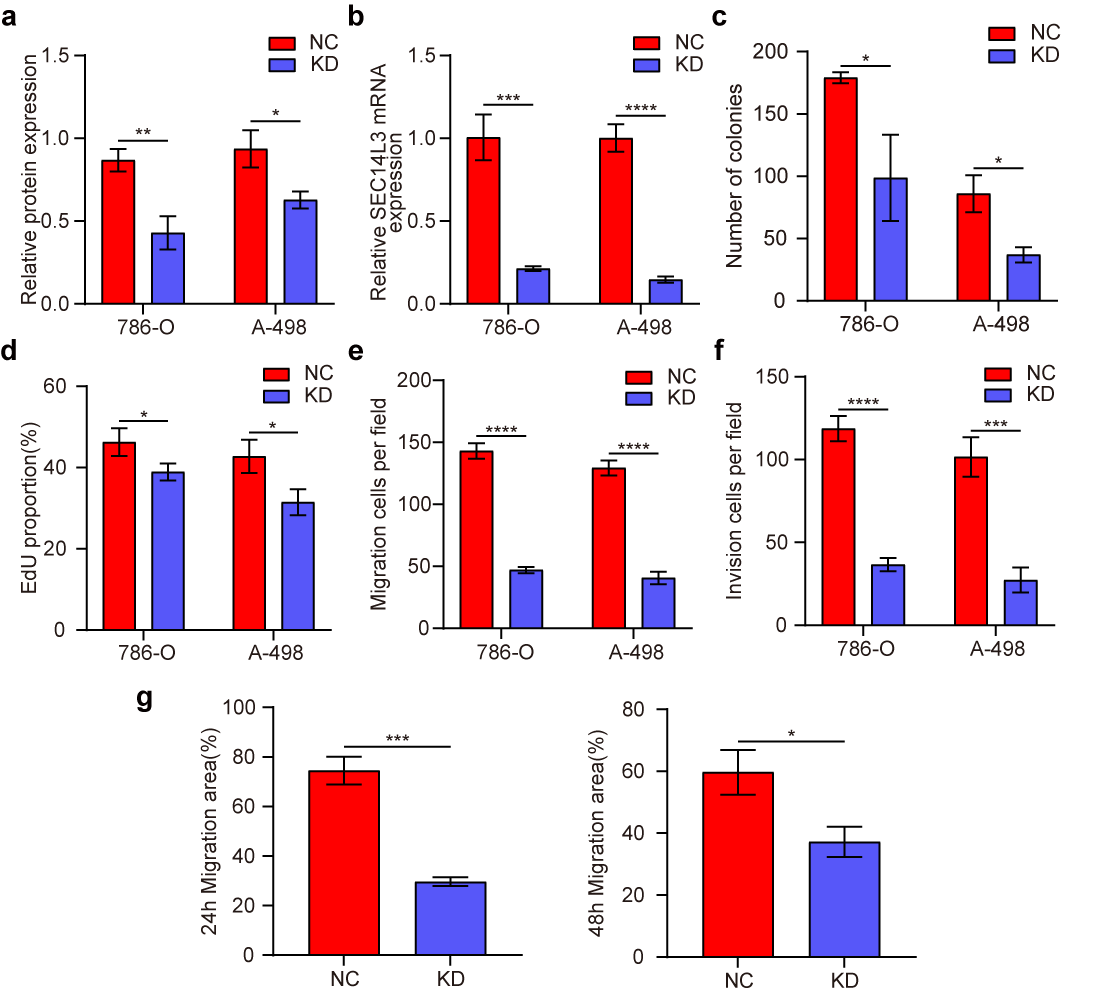

Supplement: Supplementary file 3 — Supplementary Material 3 [file 13046_2024_3206_MOESM3_ESM.tif]

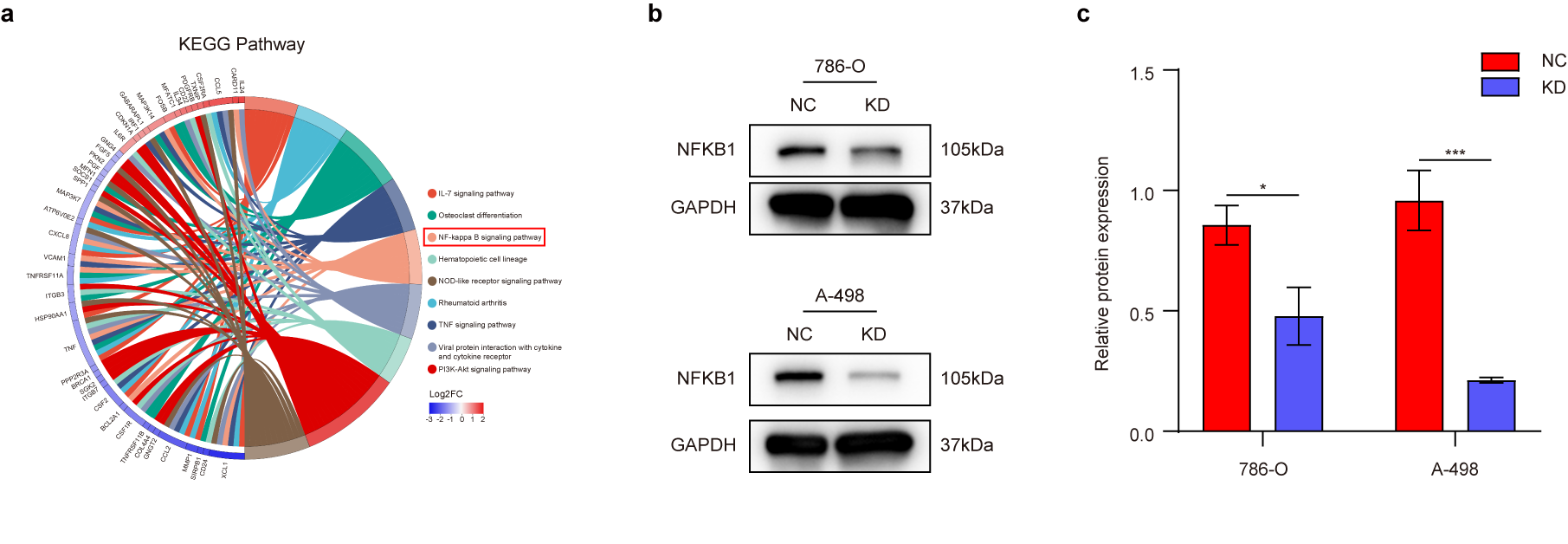

Supplement: Supplementary file 4 — Supplementary Material 4 [file 13046_2024_3206_MOESM4_ESM.tif]

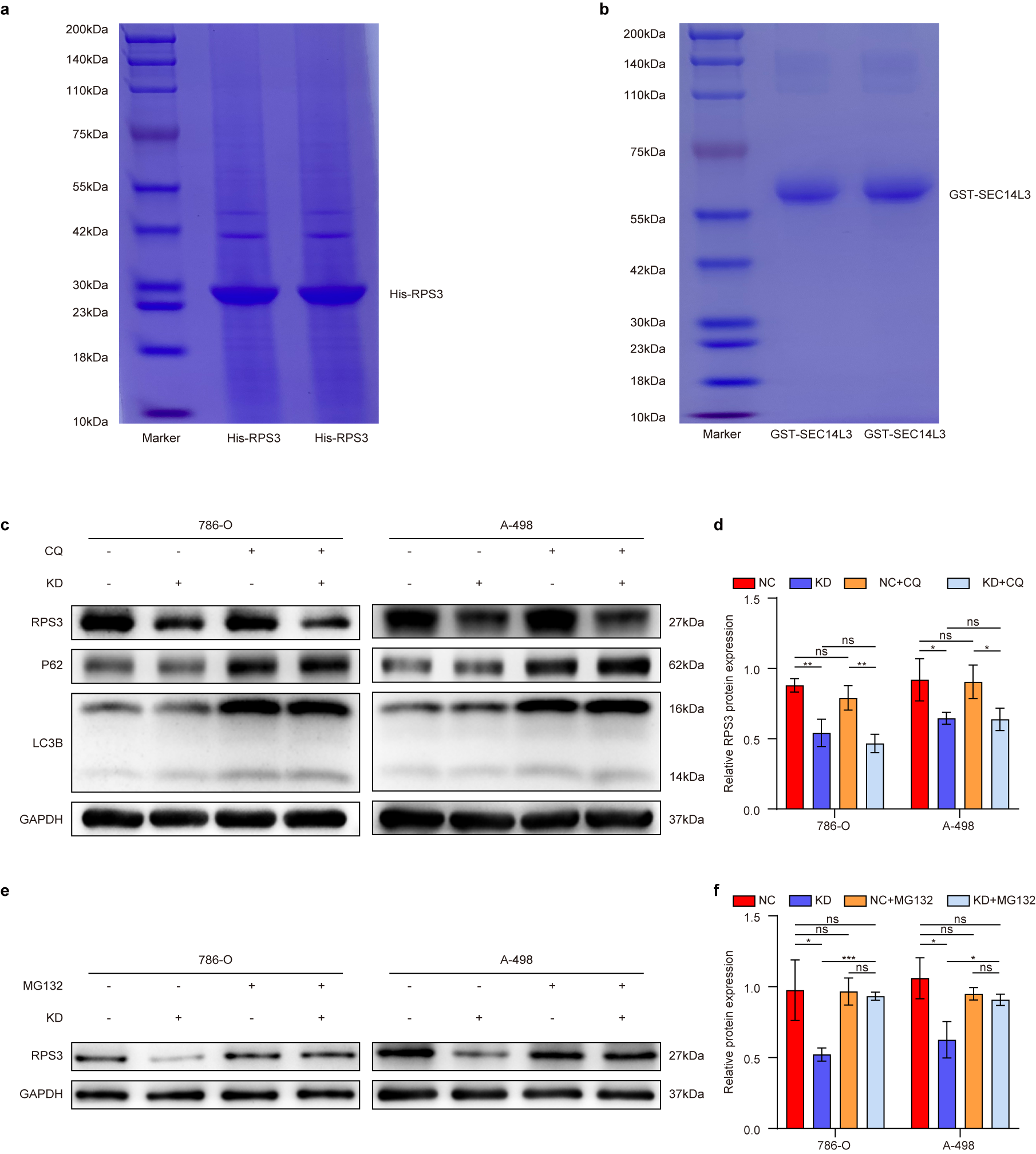

Supplement: Supplementary file 5 — Supplementary Material 5 [file 13046_2024_3206_MOESM5_ESM.tif]

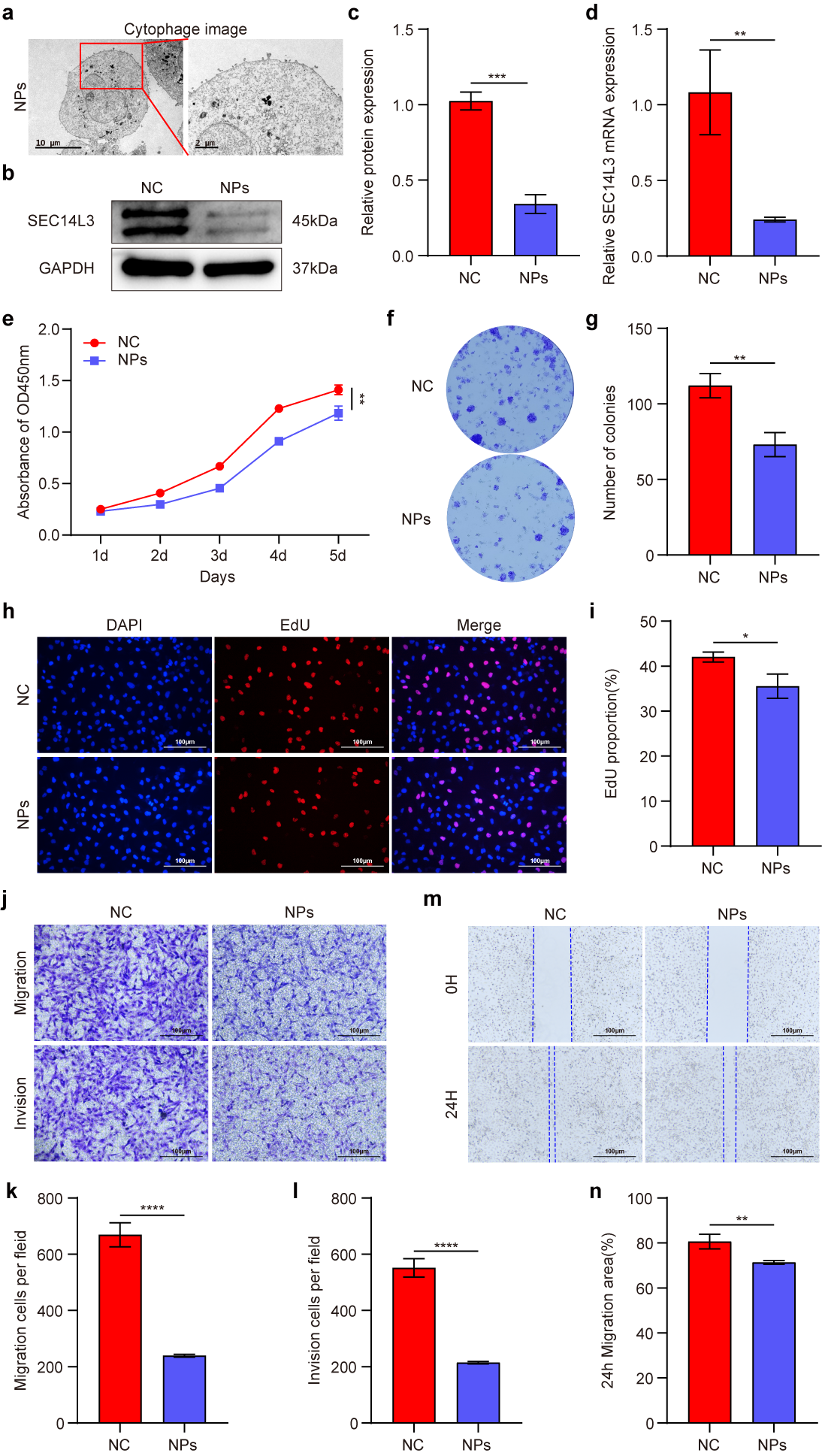

Supplement: Supplementary file 6 — Supplementary Material 6 [file 13046_2024_3206_MOESM6_ESM.tif]

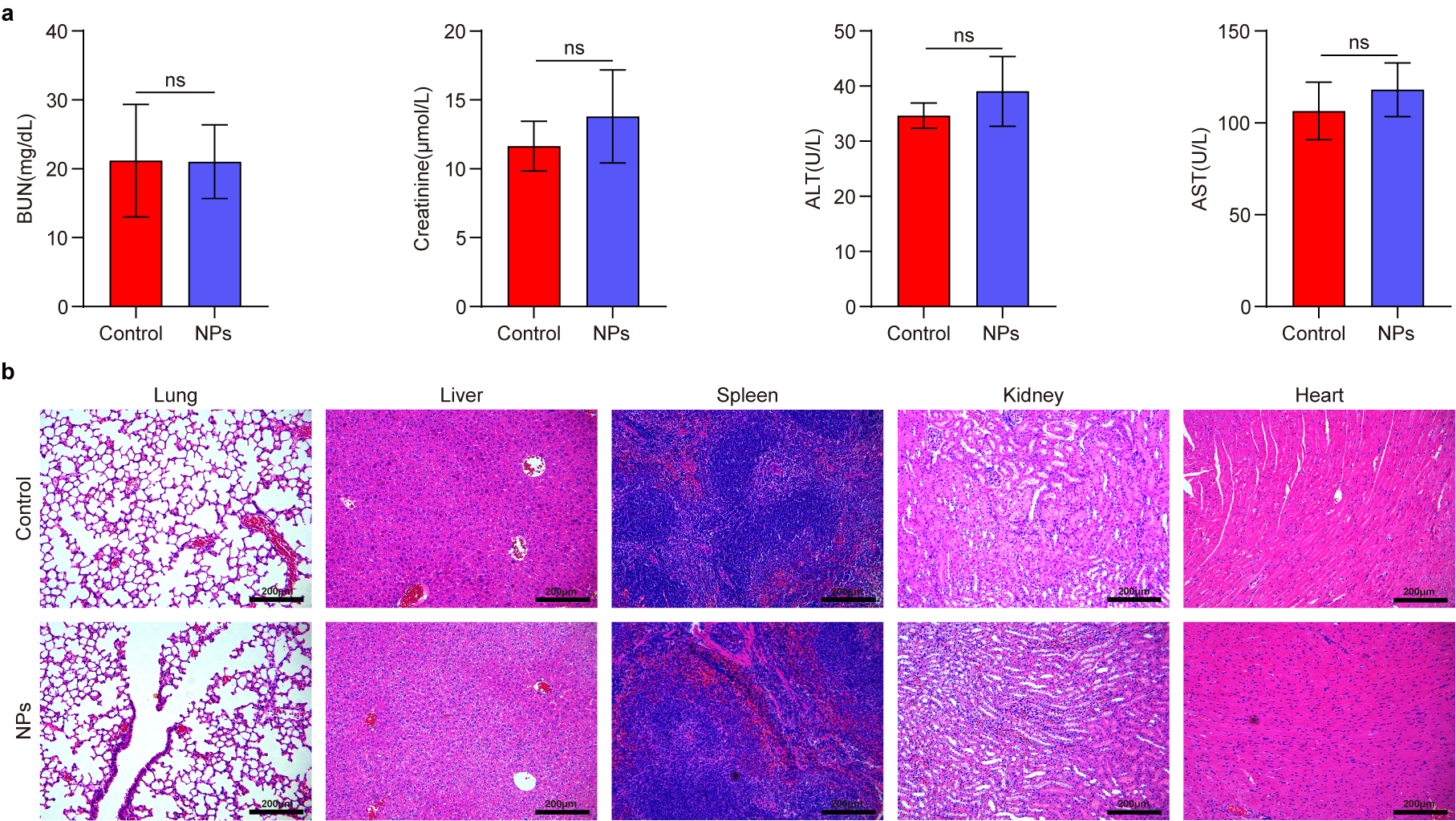

Supplement: Supplementary file 7 — Supplementary Material 7 [file 13046_2024_3206_MOESM7_ESM.tif]

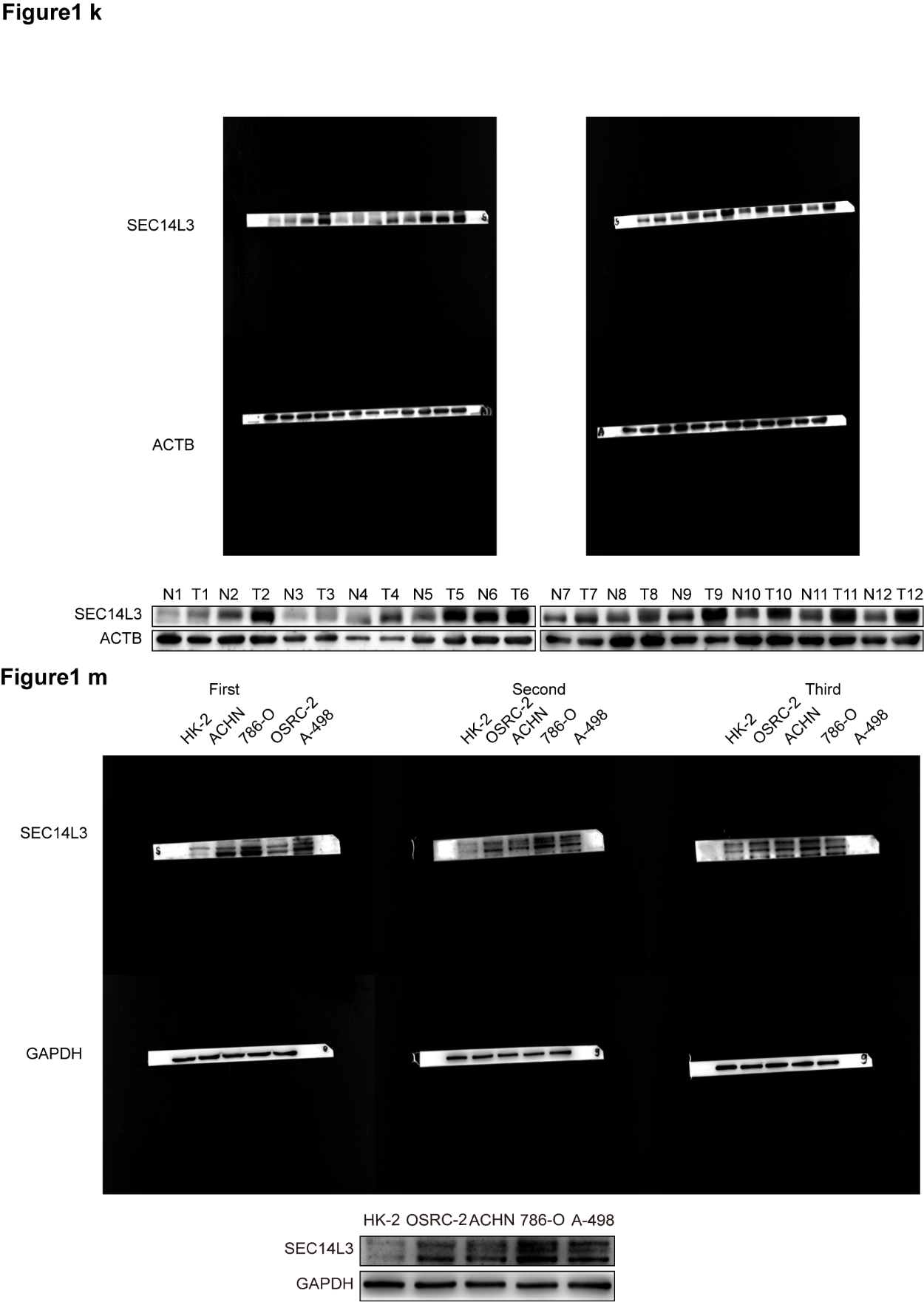


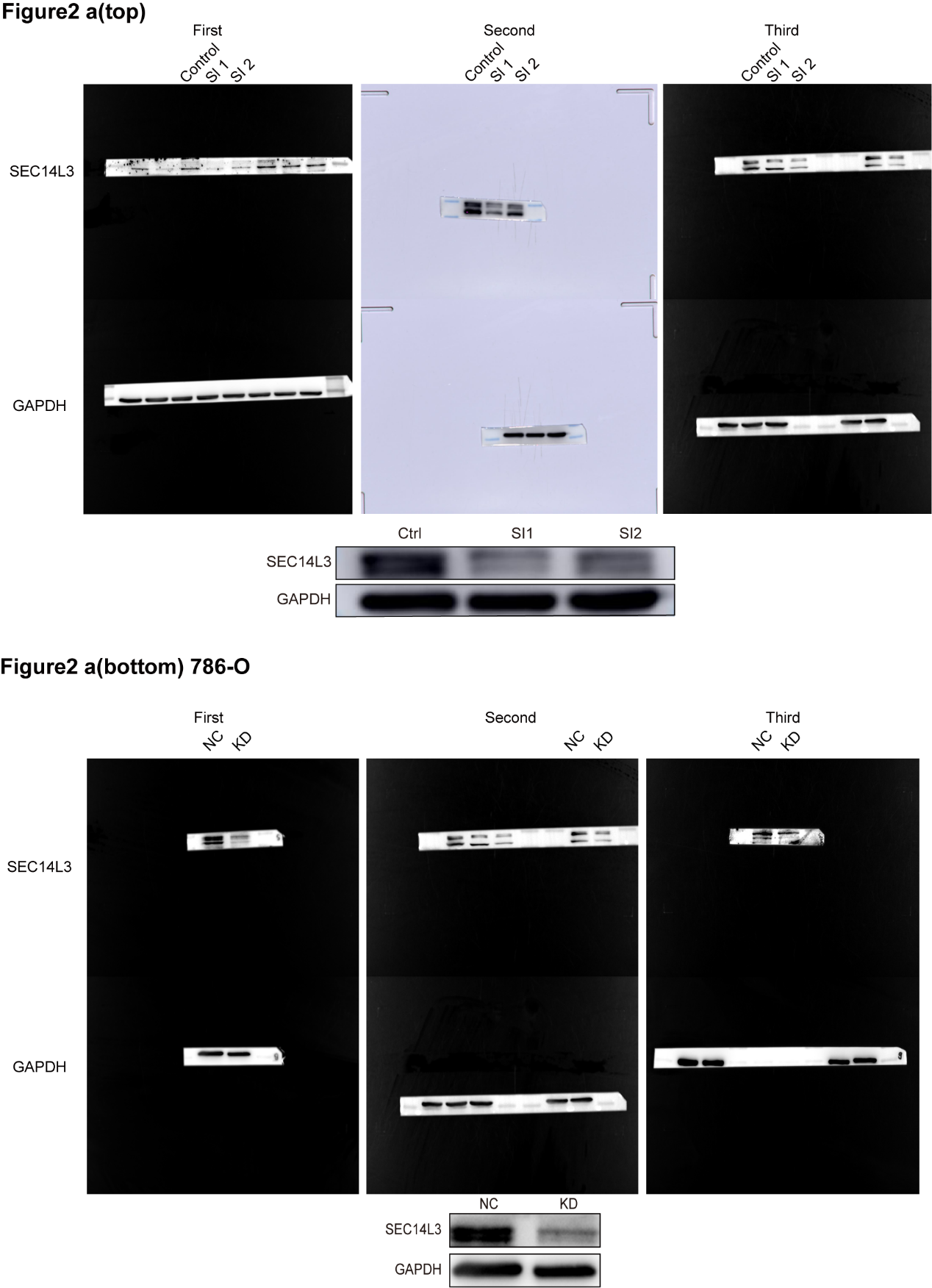


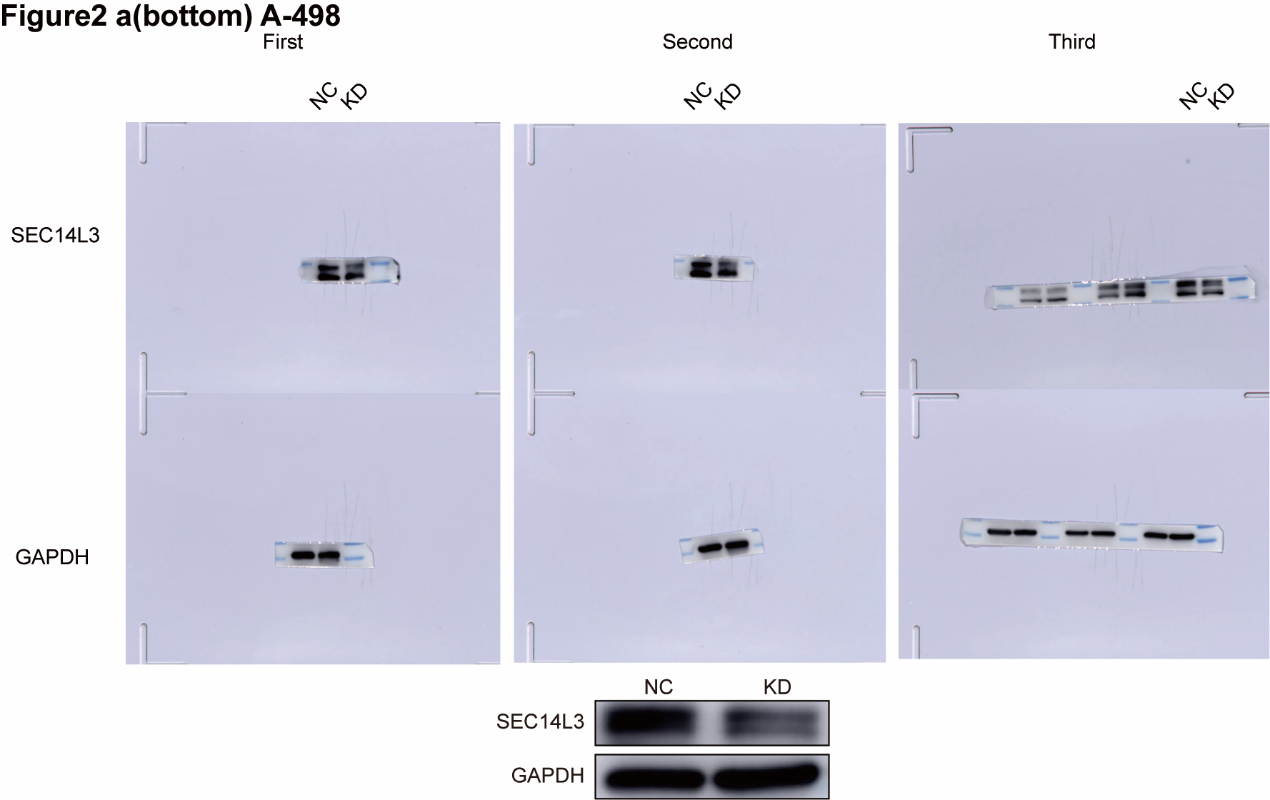

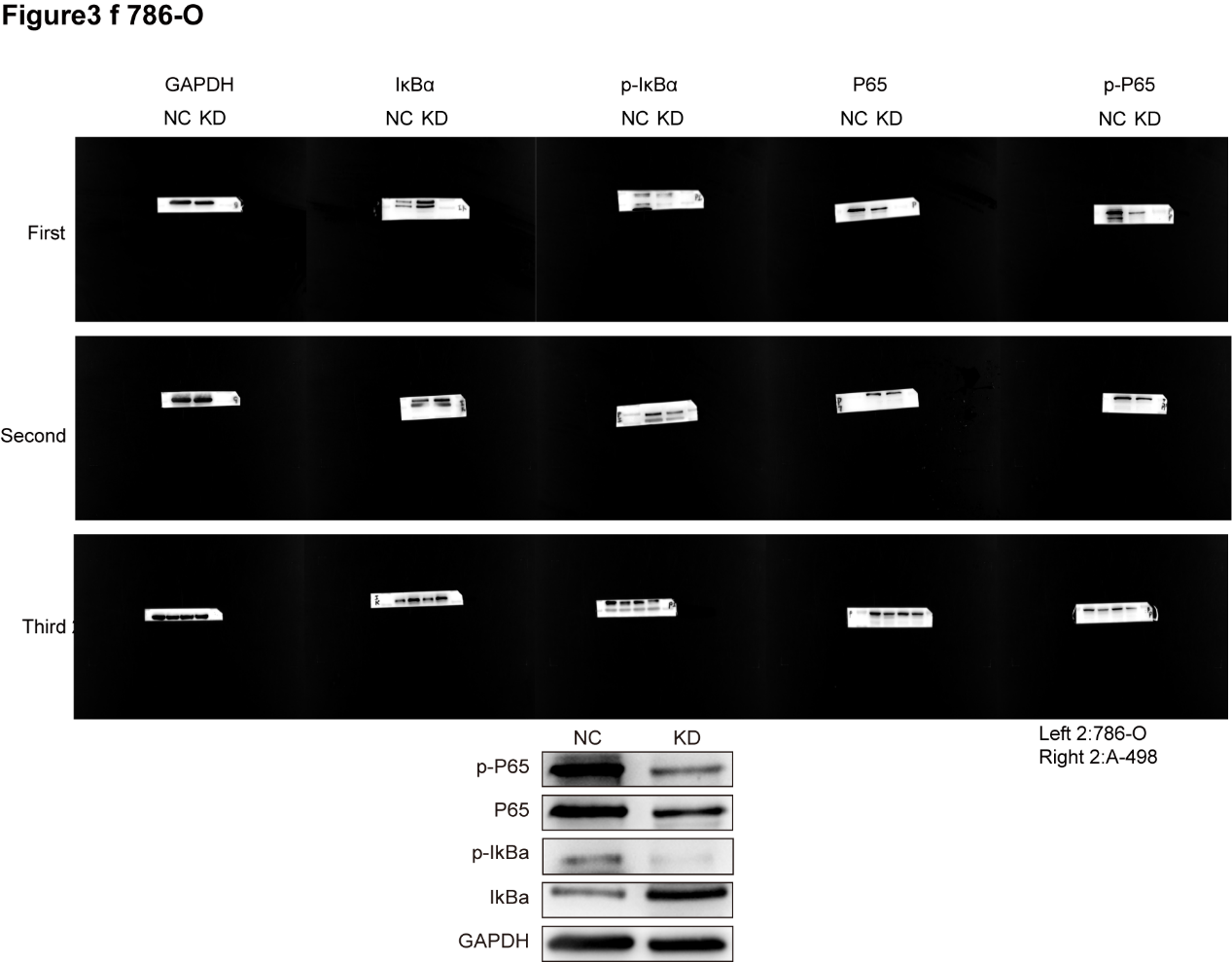


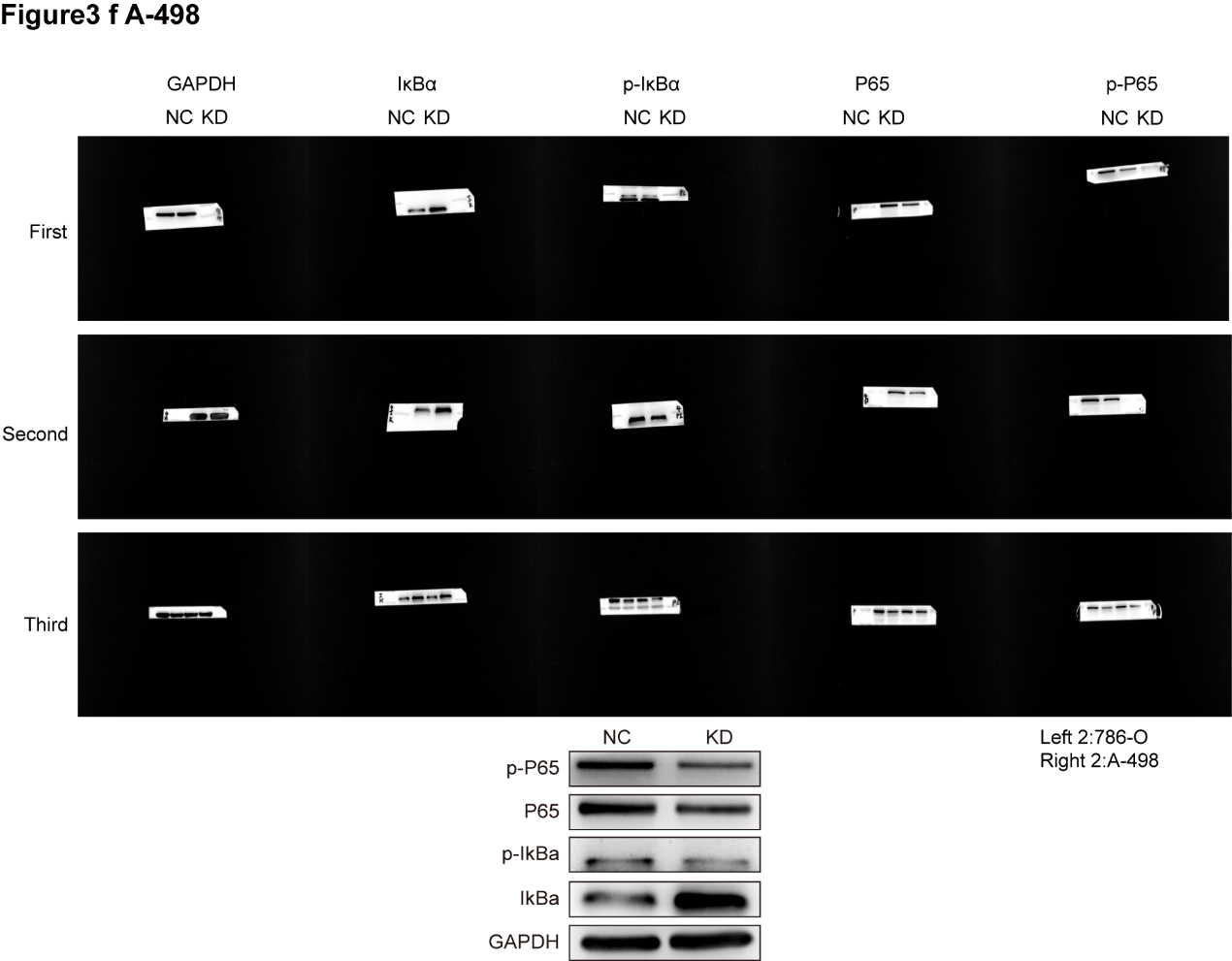

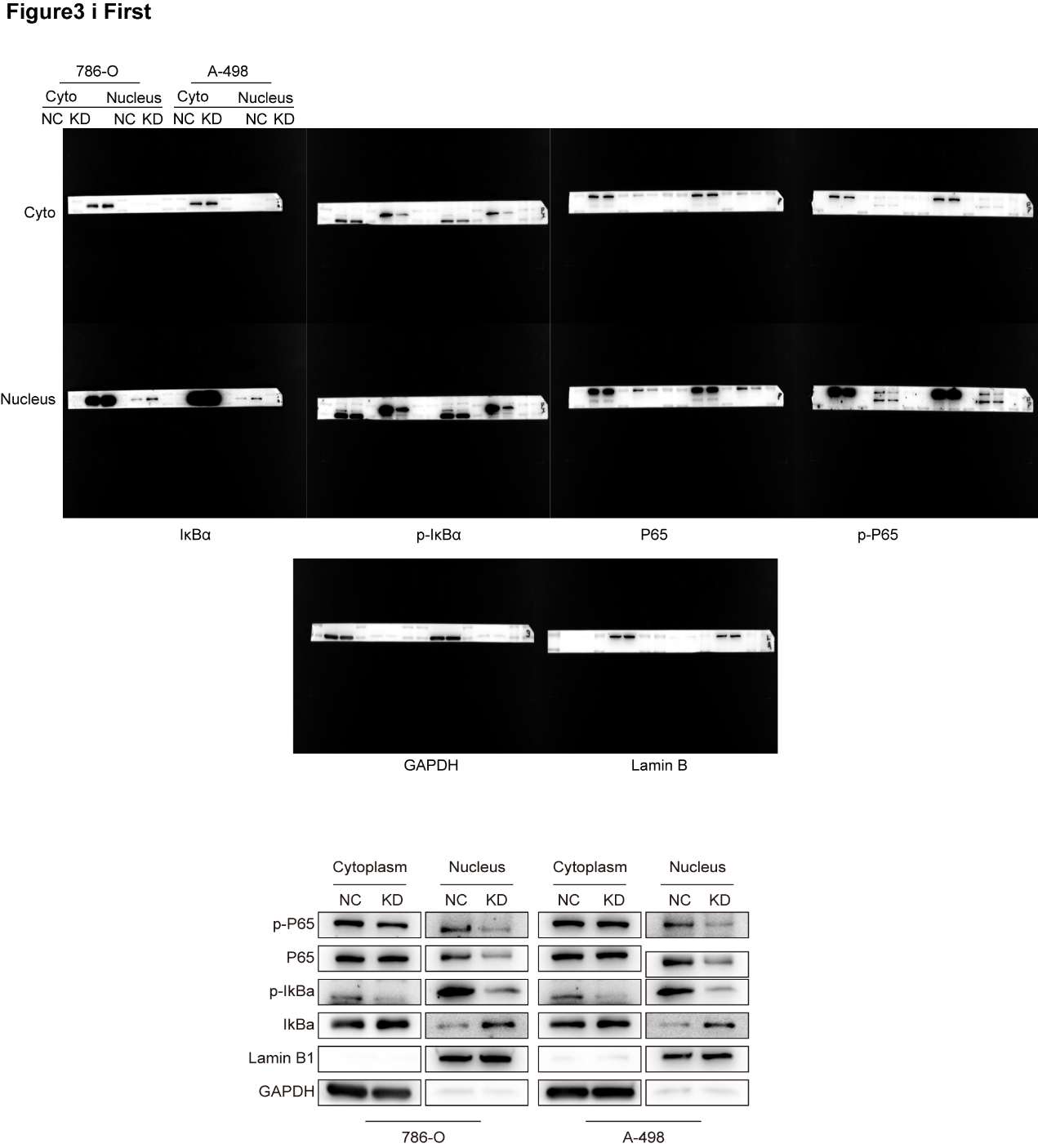


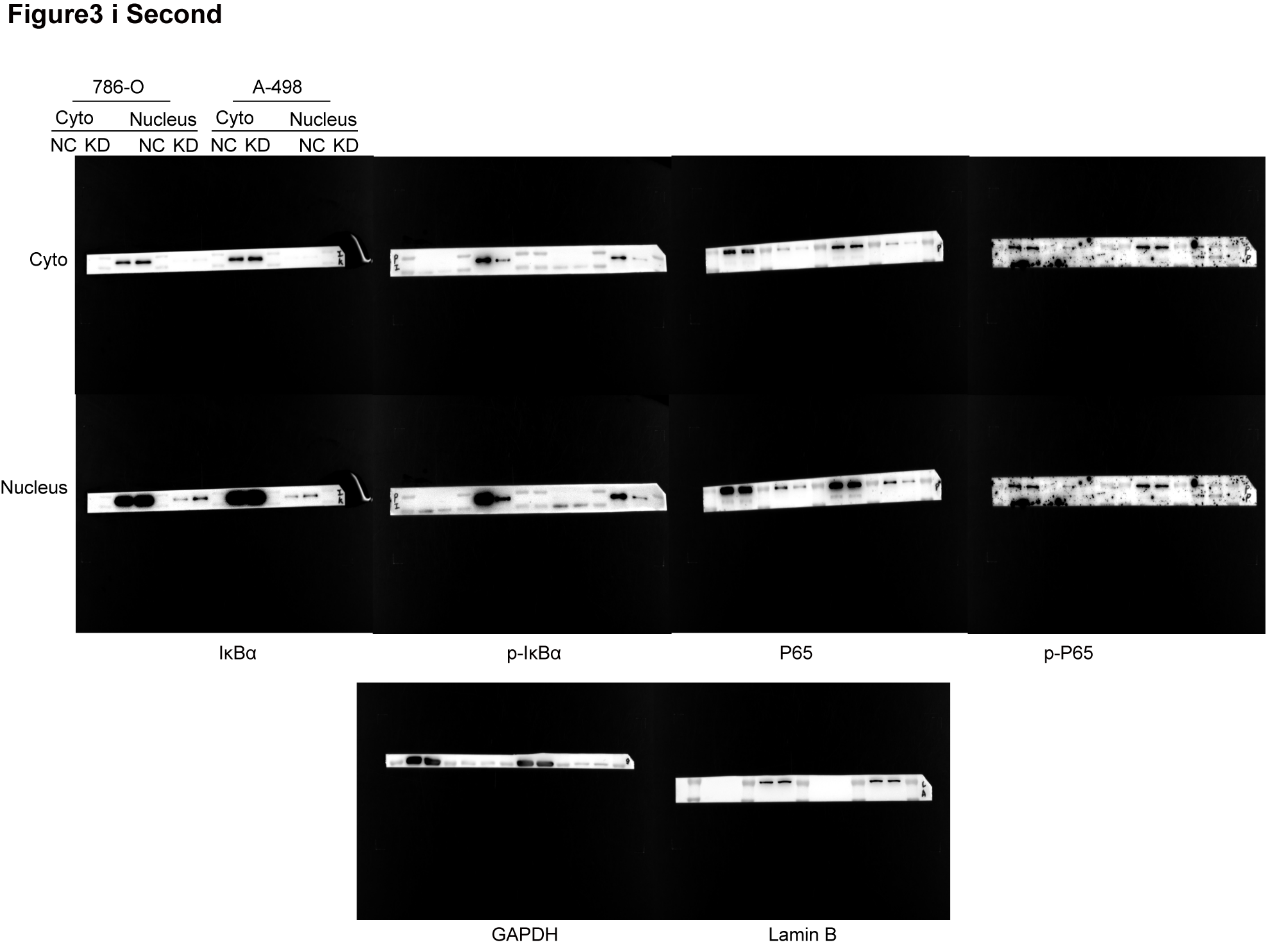

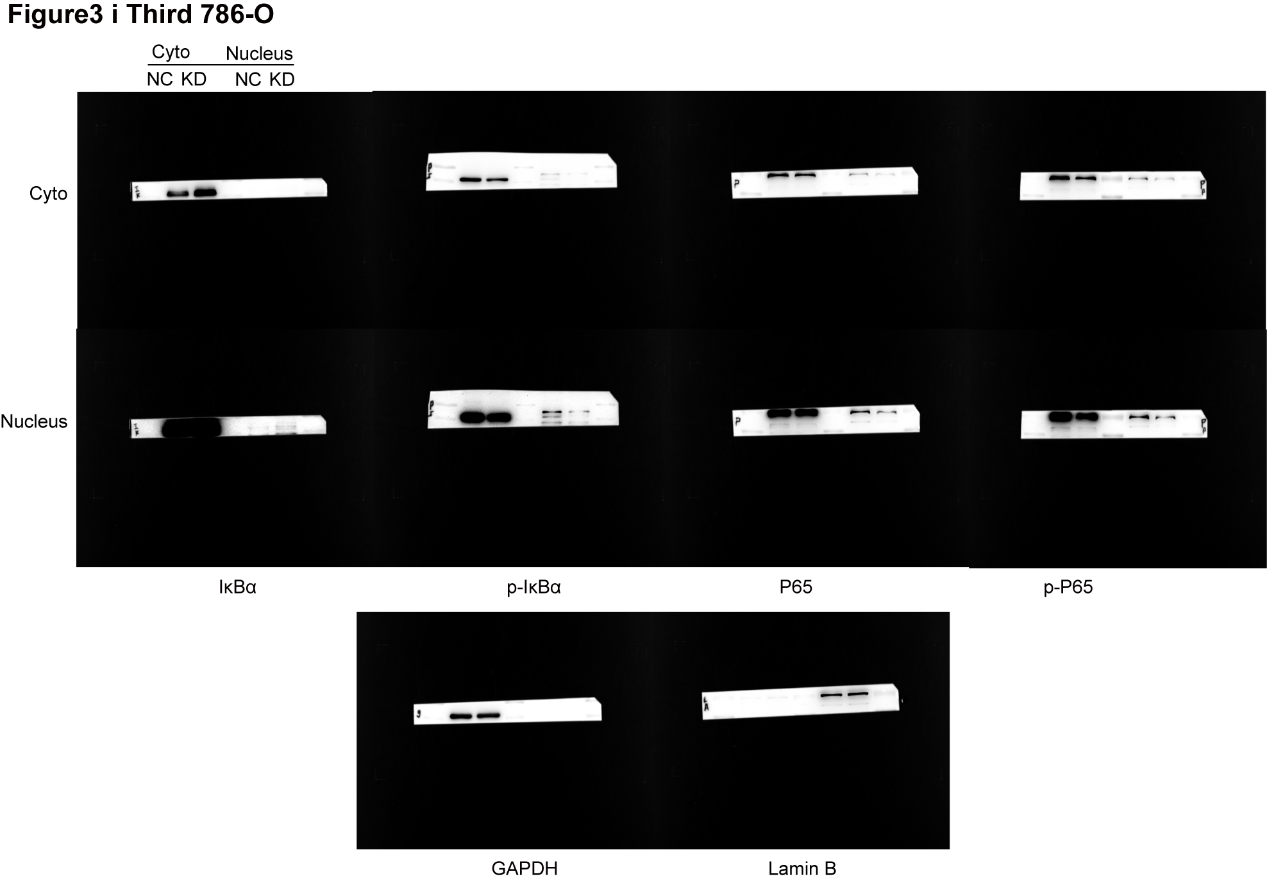


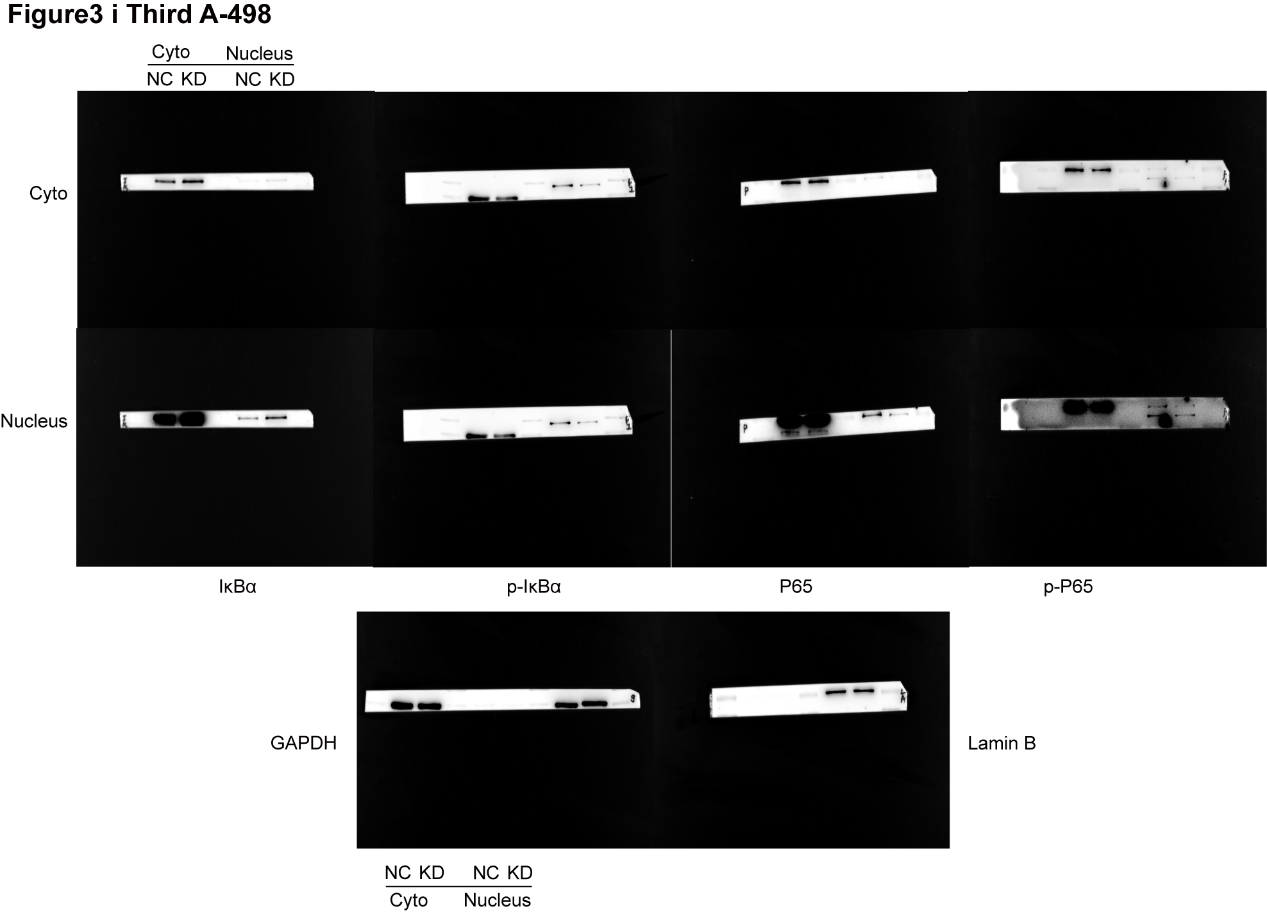

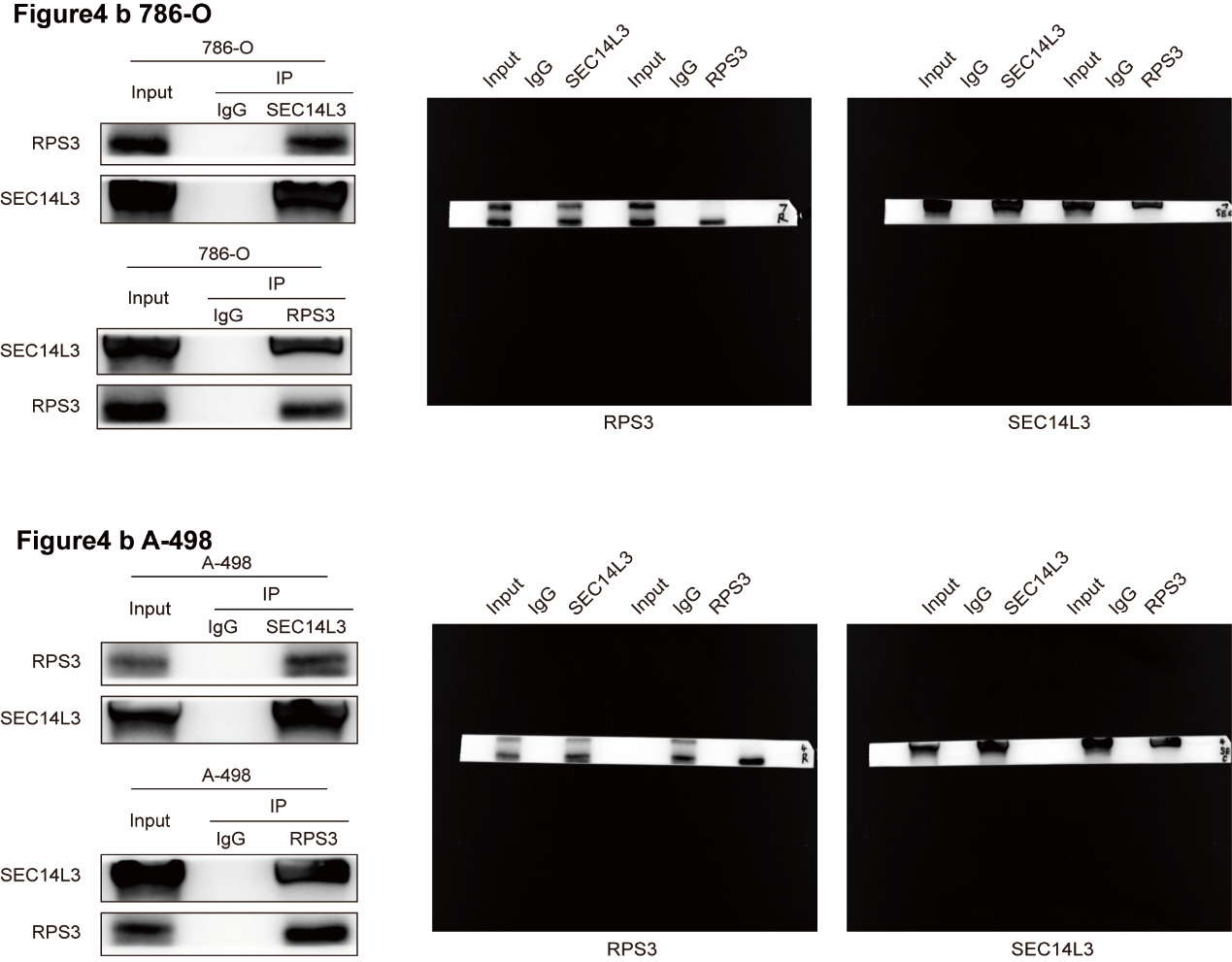


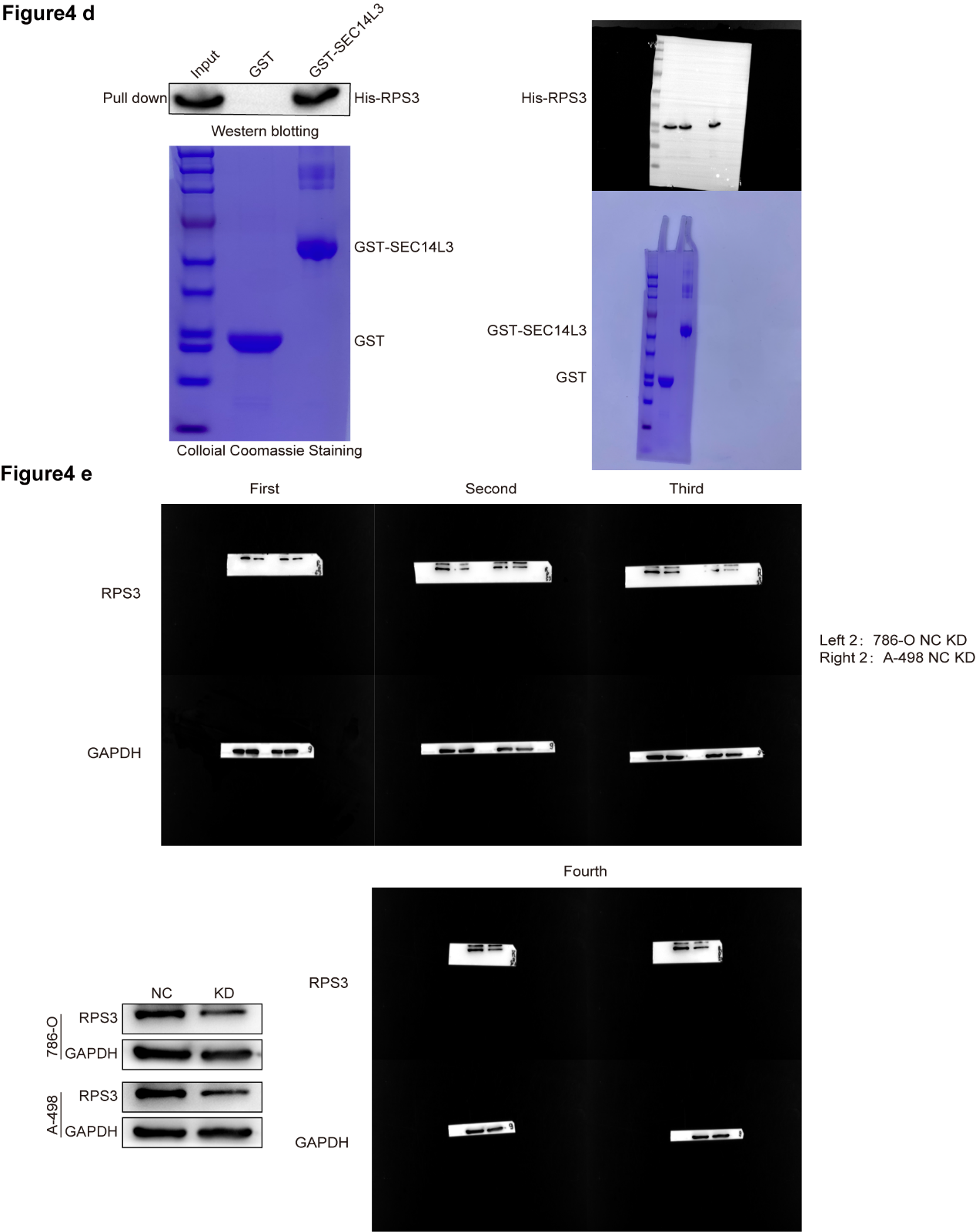

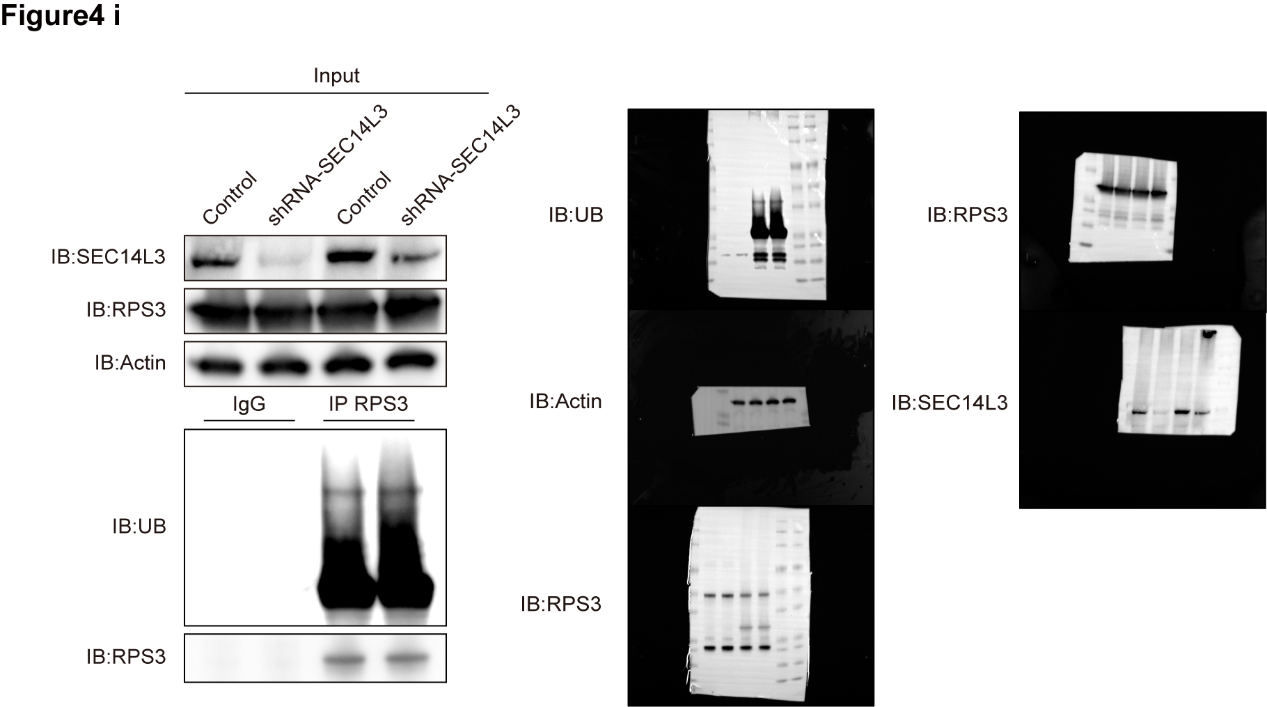


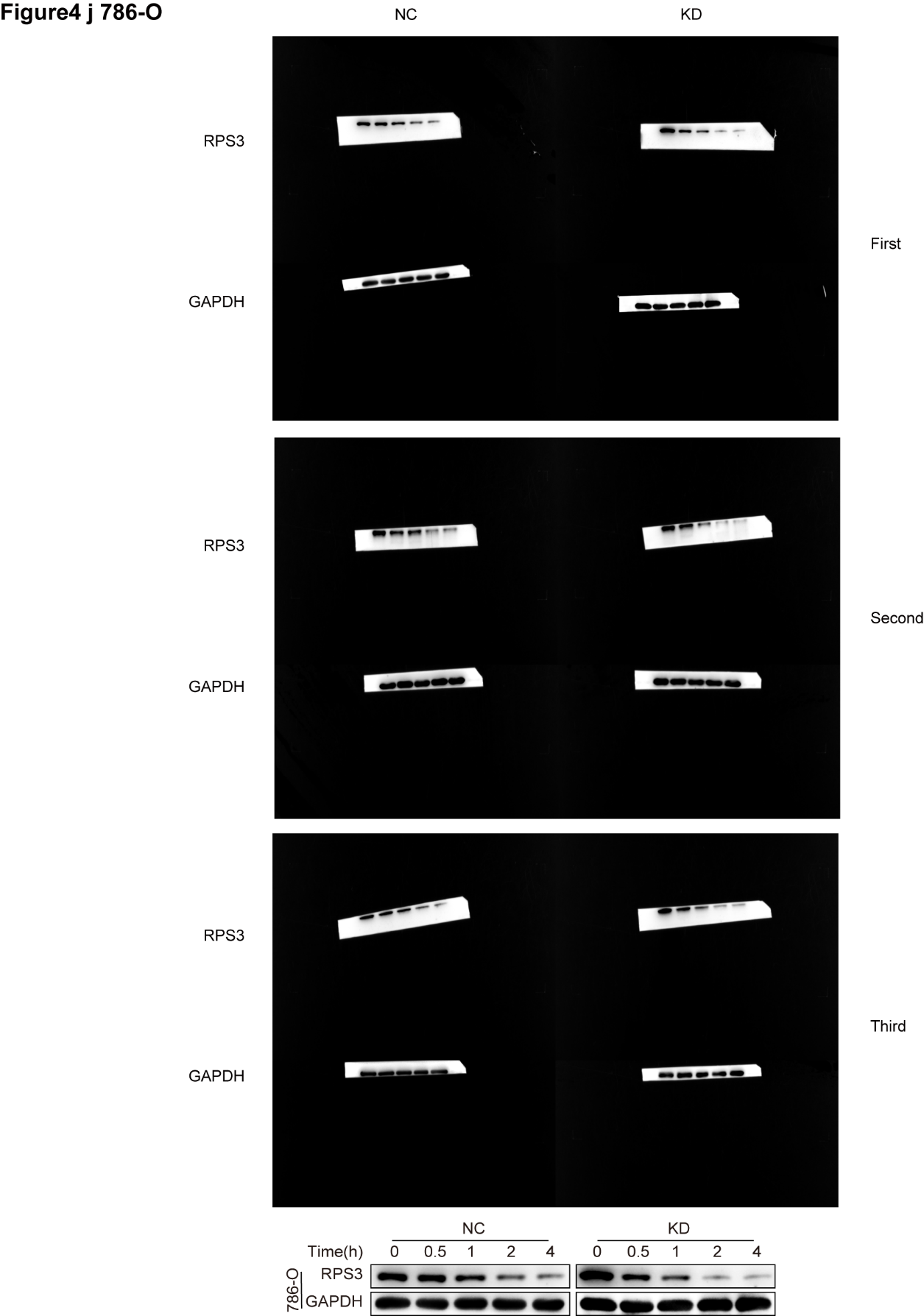

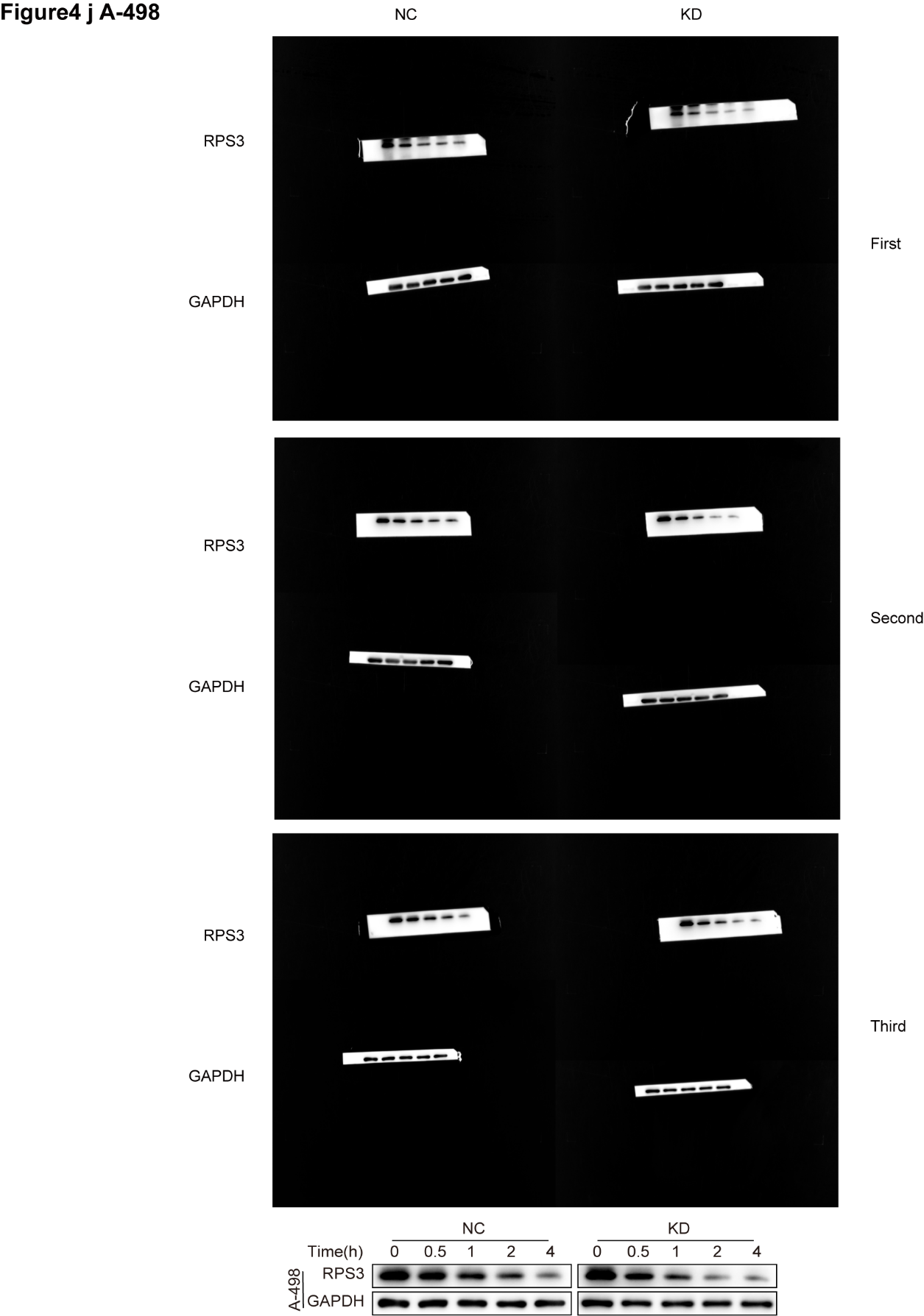


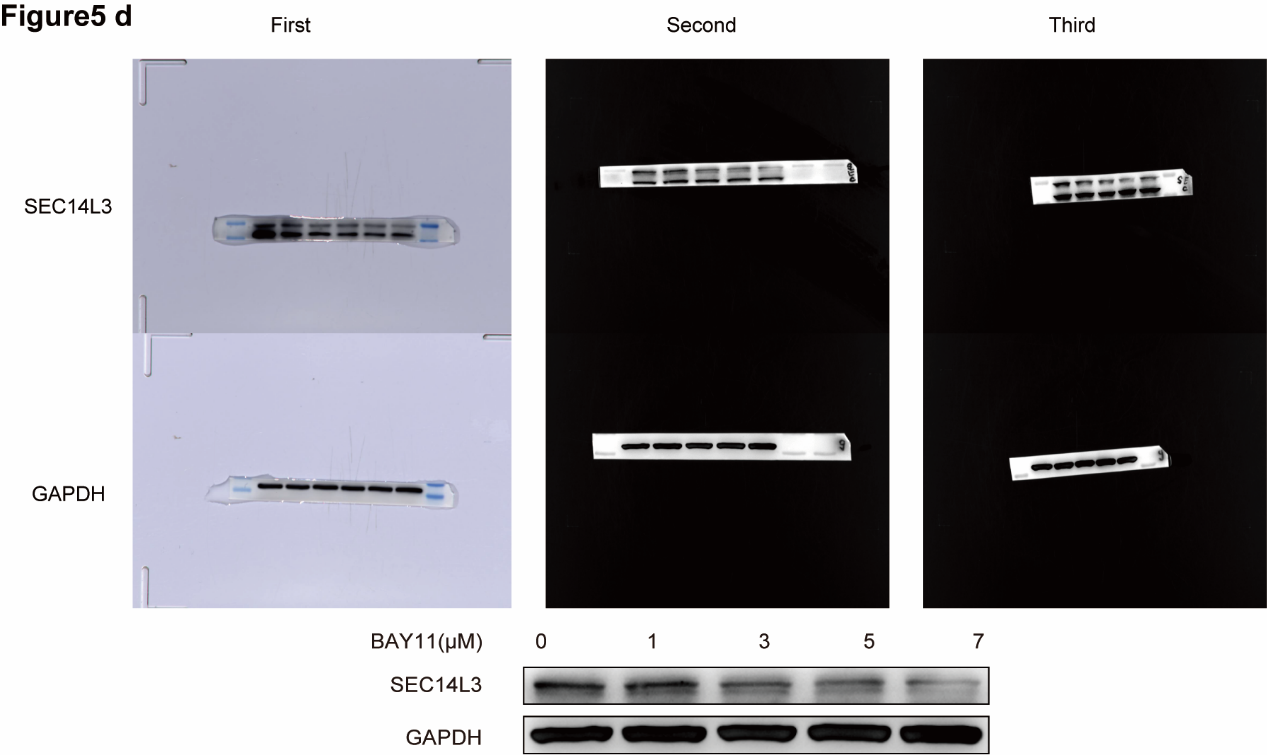

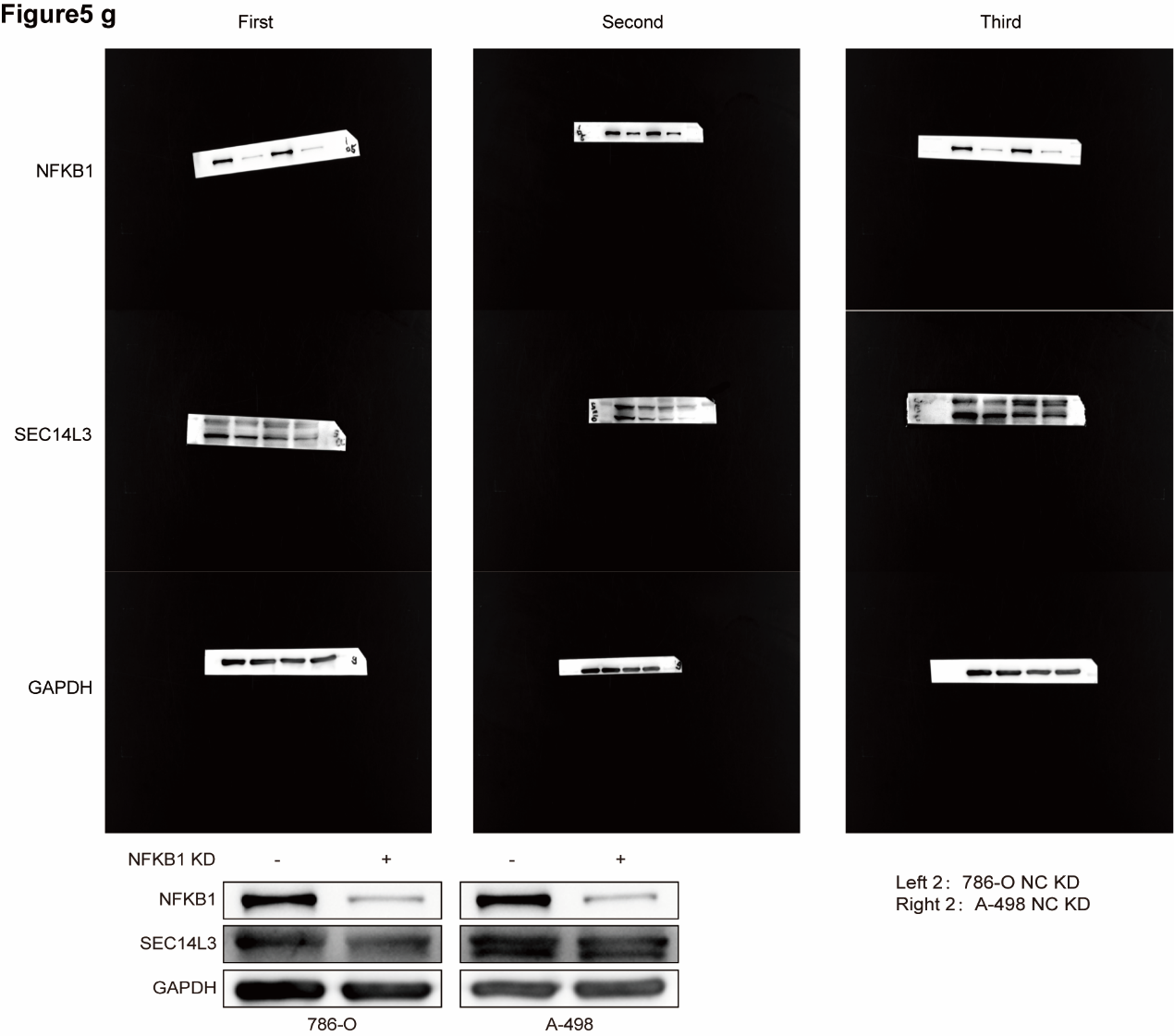


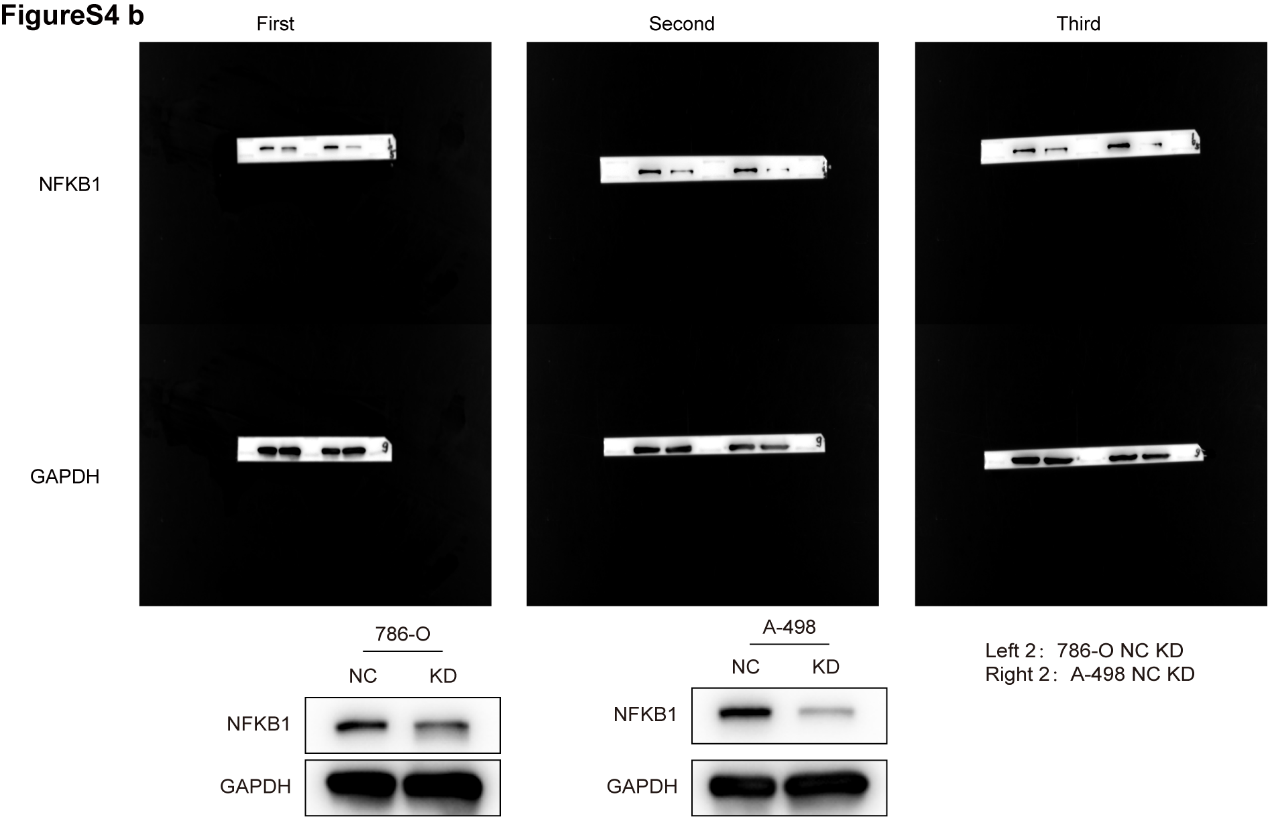

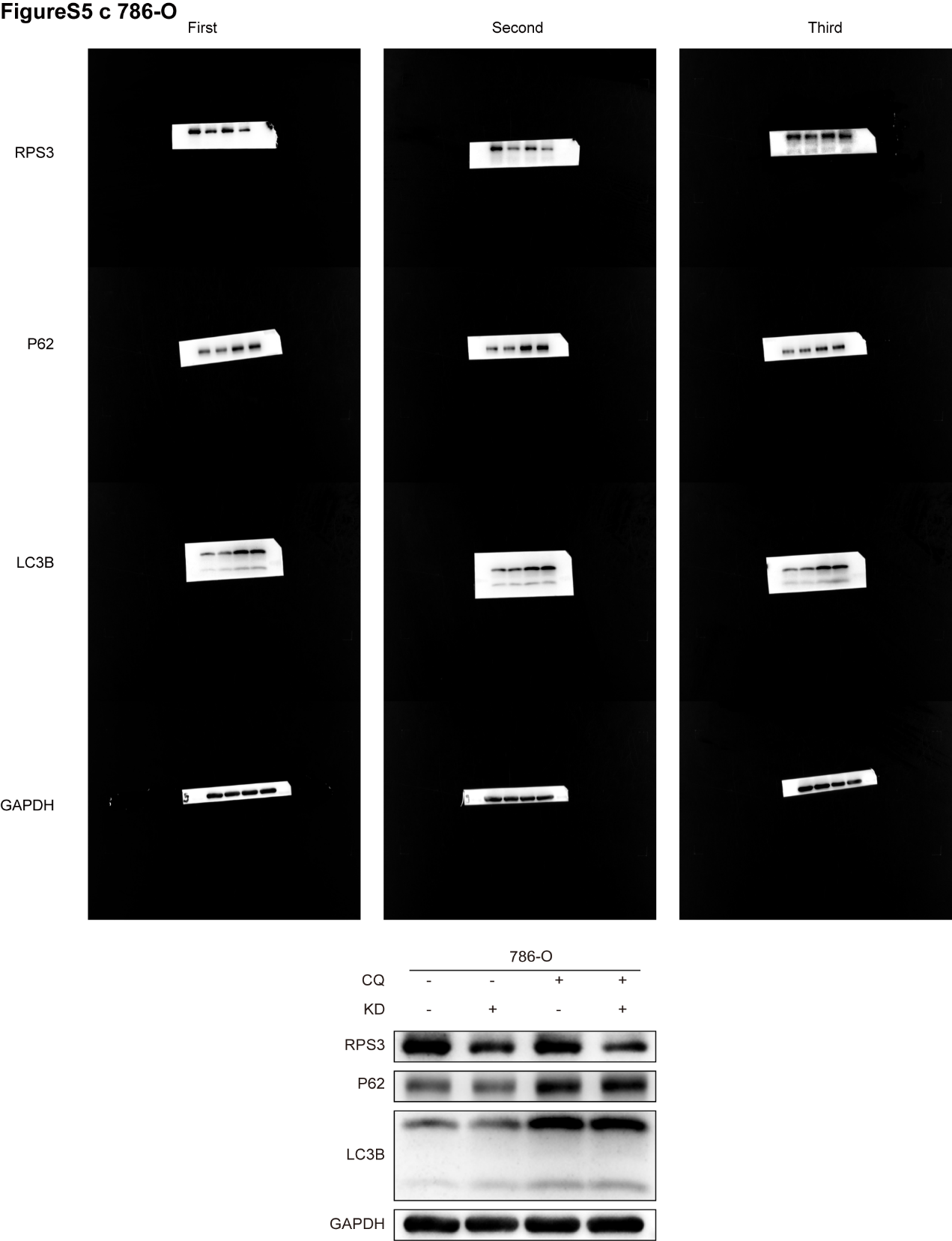


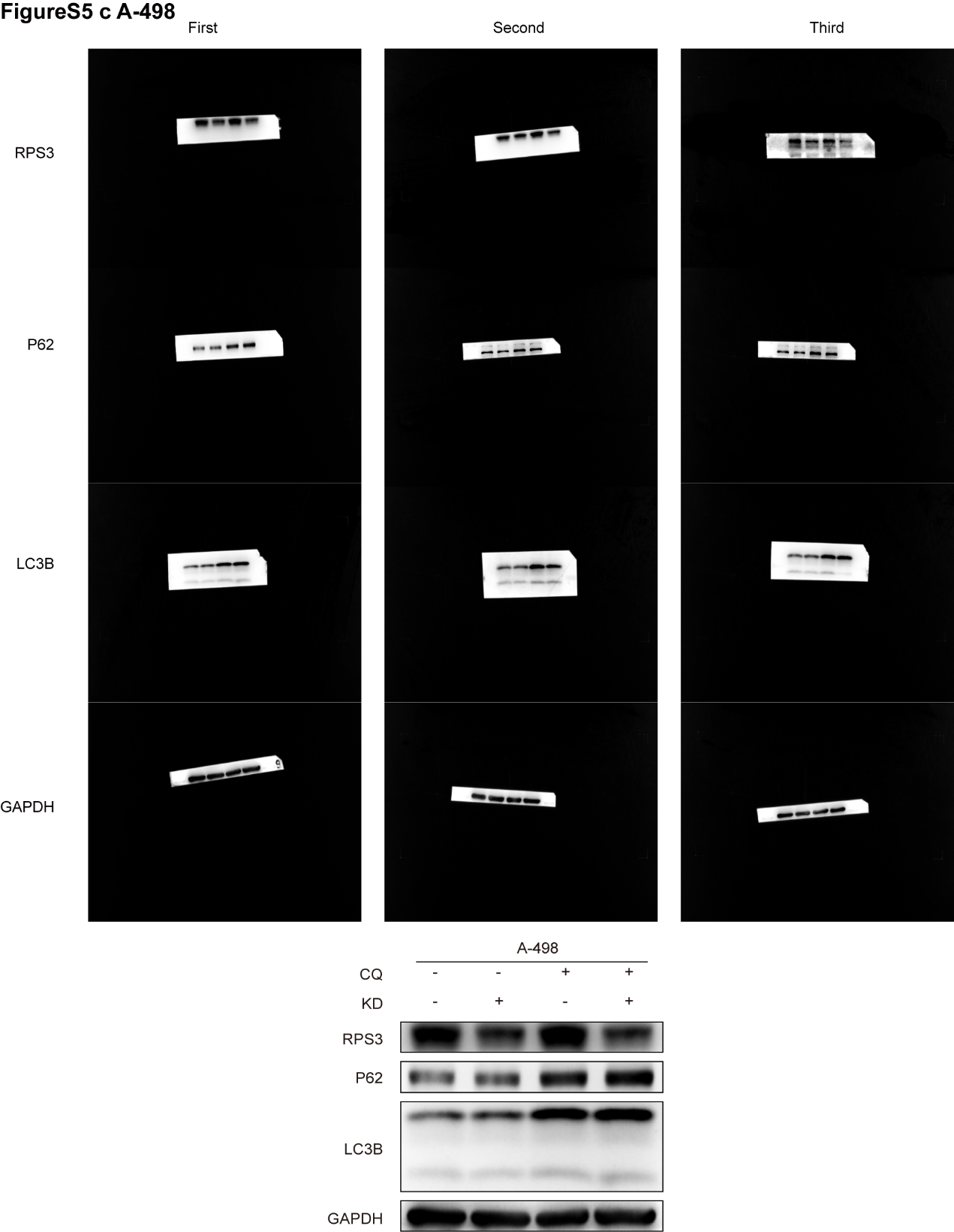

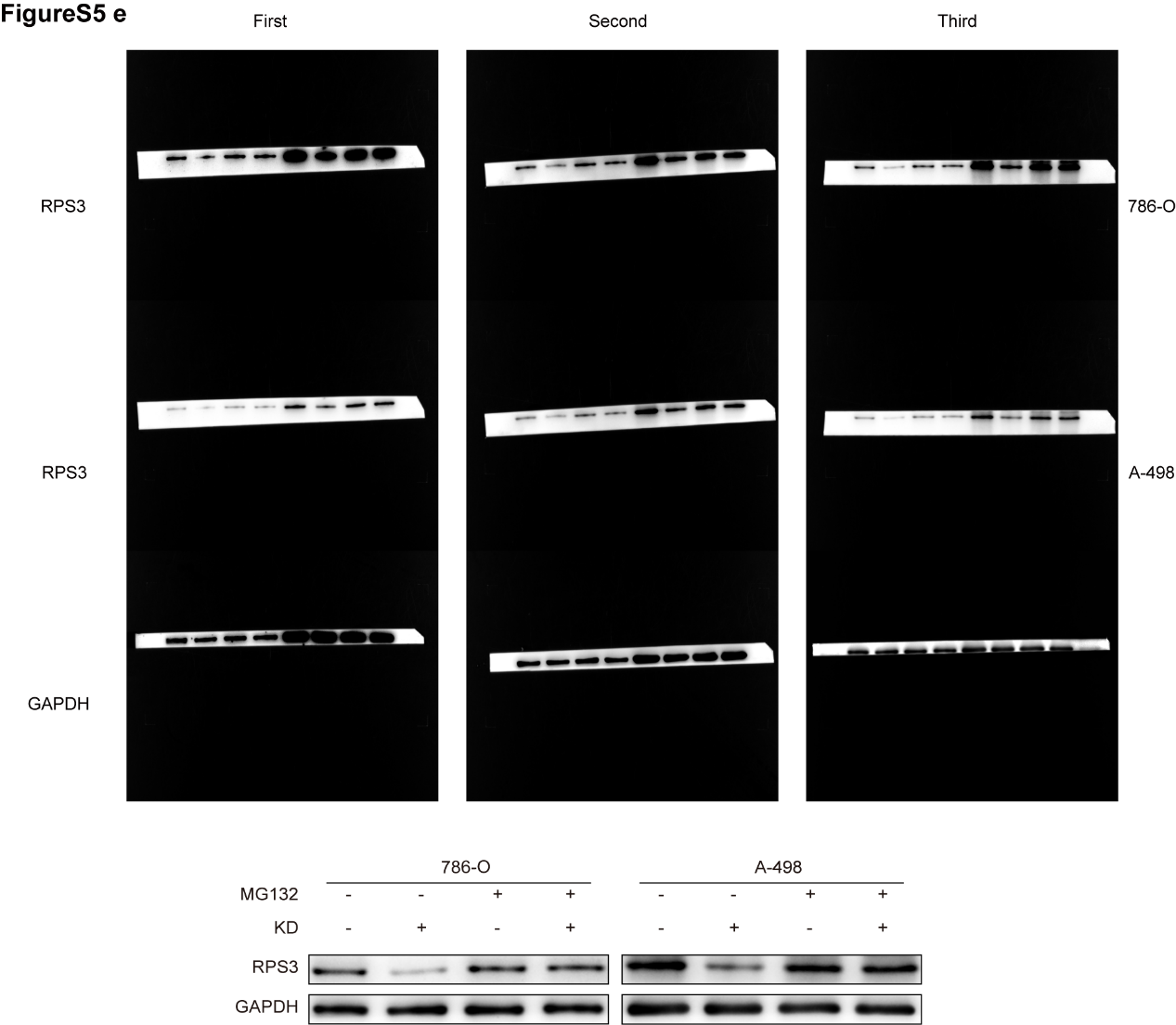


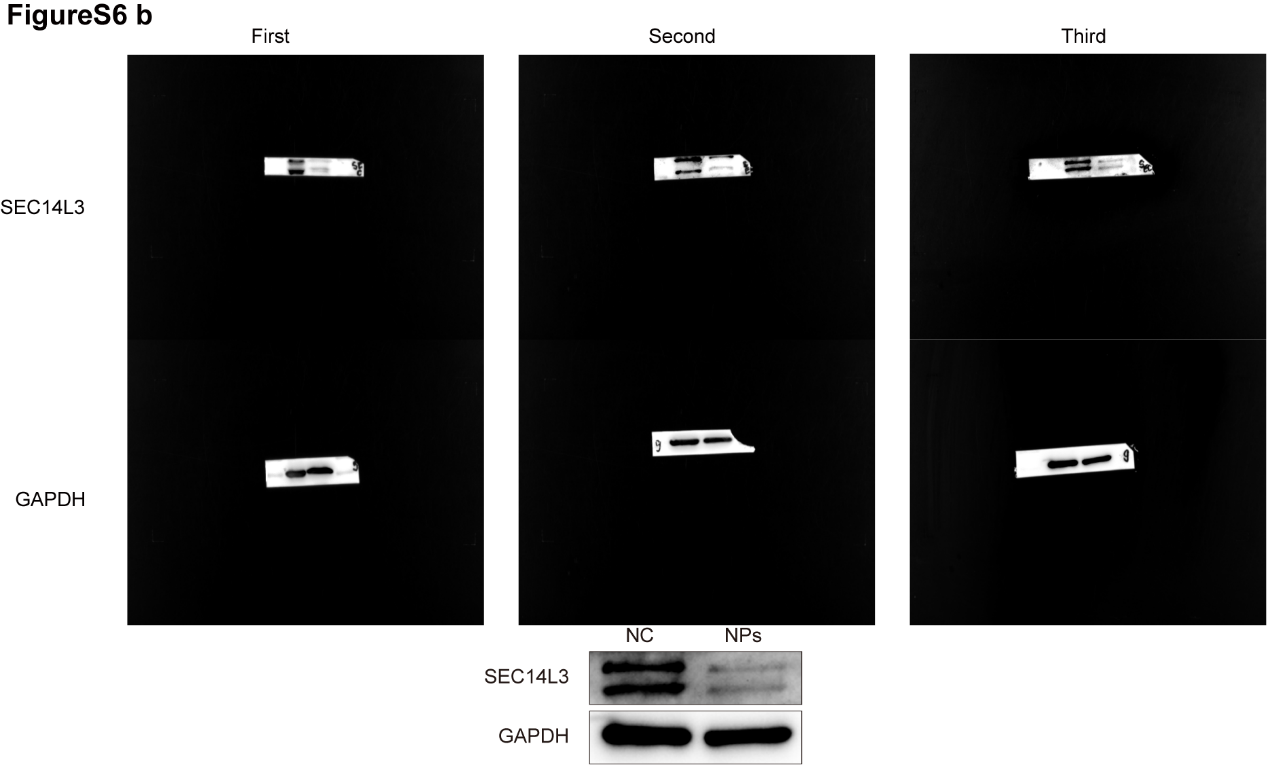

Supplement: Supplementary file 11 — Supplementary Material 11 [file 13046_2024_3206_MOESM11_ESM.docx]
