# Supplementary material for: SEC14L3 knockdown inhibited clear cell renal cell carcinoma proliferation, metastasis and sunitinib resistance through an SEC14L3/RPS3/NFκB positive feedback loop
Source: J Exp Clin Cancer Res. 2024 Oct 19;43:288. doi: 10.1186/s13046-024-03206-5 (PMC11490128; doi:10.1186/s13046-024-03206-5)
Supplement: Supplementary file 8 — Supplementary Material 8 [file 13046_2024_3206_MOESM8_ESM.docx]

**Table S1. Sequences of primers used for RT-qPCR**

| **Species** | **Gene** | **Forward (5’-3’)** | **Reverse (5’-3’)** |
| --- | --- | --- | --- |
| Human | *GAPDH* | GTCTCCTCTGACTTCAACAGCG | ACCACCCTGTTGCTGTAGCCAA |
| Human | *SEC14L3* | CGGCTCATCACACCAAGTGGAA | GGAAAACTCCGAAGCCGATGTC |
| Human | *RPS3* | GCTGAAGATGGCTACTCTGGAG | ACAGCAGTCAGTTCCCGAATCC |
| Human | *SEC14L3-promoter1* | CCACACCTGGCTAATTTTTG | GGATGGGTGGATGAAAGAAA |
| Human | *SEC14L3-promoter2* | TGTGGGCCATTTTGCATGTG | AATCATCAGGGTTGGGCAGG |
| Human | *SEC14L3-Enhencer1* | GGTGGATCACAAGGTCAGGA | GCAACTTCCACCTCATGGTT |
| Human | *SEC14L3-Enhencer2* | TCATGGTGATTAGGCAGAGGG | AGTCATGAGCAGTGAAACTAAGA |

**Table S2. Sequences of shRNAs and siRNAs**

**Sequences for siRNAs:**

| siSEC14L3#1 | 5’-GACTGAAACACTTCTGGAA-3’ |
| --- | --- |
| siSEC14L3#2 | 5’-GAAGGTTTGCTGAAACTCA-3’ |

| shSEC14L3 | 5’-CCGGCGTGAAAGCTACCAAACTGTTCTCGAGAACAGTTTGGTAGCTTTCACGTTTTTG-3’ |
| --- | --- |

**Sequences of shRNAs:**

**Sequences of lentiviruses**

| SEC14L3 knockdown | 5’-CCGGGACTGAAACACTTCTGGAACTCGAGTTCCAGAAGTGTTTCAGTCTTTTTG-3’ |
| --- | --- |
| NFKB1 knockdown | 5’-CCGGCCTTTCCTCTACTATCCTGAACTCGAGTTCAGGATAGTAGAGGAAAGGTTTTTG-3’ |

**Table S3. Antibodies**

| **Gene** | **Catalog Number** | **Company** | **Dilution** |
| --- | --- | --- | --- |
| SEC14L3 | #ab235110 | Abcam | 1:3000 WB |
| RPS3 | #11990-1-AP | Proteintech | 1:1000 WB |
| NFKB1 | #AY9315 | Abways | 1:1000 WB |
| P65 | #T55034 | Abmart | 1:5000 WB |
| p-P65 | #TP56372 | Abmart | 1:1000 WB, 1:200 IHC |
| IκBα## | #T55026 | Abmart | 1:1000 WB |
| p-IκBα | #TP56280 | Abmart | 1:1000 WB |
| Lamin B1 | #AB0054 | Abways | 1:10000 WB |
| LC3B | #AB192890 | Abcam | 1:1000 WB |
| P62 | #AB109012 | Abcam | 1:1000 WB |
| Ki-67 | #TA0198 | Abmart | 1:200 IHC |
| Ubiquitin | #10201-2-AP | Proteintech | 1:1000 WB |
| Flag | #66008-4-Ig | Proteintech | 1:5000 WB |
| HA | #HRP-81290 | Proteintech | 1:5000 WB |
| His | #66005-1-Ig | Proteintech | 1:5000 WB |
| Gapdh | #A19056 | ABclonal | 1:50000 WB |
| Actin | #AC038 | ABclonal | 1:10000 WB |

**Table S4. Sequences for His-RPS3 and GST-SEC14L3**

| His-RPS3 |
| --- |
| 5’-ATGGGCAGCAGCCATCATCATCATCATCACAGCAGCGGCCTGGTGCCGCGCGGCAGCCATATGGCTAGCATGACTGGTGGACAGCAAATGGGTCGCGGATCCATGGCAGTGCAAATATCCAAGAAGAGGAAGTTTGTCGCTGATGGCATCTTCAAAGCTGAACTGAATGAGTTTCTTACTCGGGAGCTGGCTGAAGATGGCTACTCTGGAGTTGAGGTGCGAGTTACACCAACCAGGACAGAAATCATTATCTTAGCCACCAGAACACAGAATGTTCTTGGTGAGAAGGGCCGGCGGATTCGGGAACTGACTGCTGTAGTTCAGAAGAGGTTTGGCTTTCCAGAGGGCAGTGTAGAGCTTTATGCTGAAAAGGTGGCCACTAGAGGTCTGTGTGCCATTGCCCAGGCAGAGTCTCTGCGTTACAAACTCCTAGGAGGGCTTGCTGTGCGGAGGGCCTGCTATGGTGTGCTGCGGTTCATCATGGAGAGTGGGGCCAAAGGCTGCGAGGTTGTGGTGTCTGGGAAACTCCGAGGACAGAGGGCTAAATCCATGAAGTTTGTGGATGGCCTGATGATCCACAGCGGAGACCCTGTTAACTACTACGTTGACACTGCTGTGCGCCACGTGTTGCTCAGACAGGGTGTGCTGGGCATCAAGGTGAAGATCATGCTGCCCTGGGACCCAACTGGTAAGATTGGCCCTAAGAAGCCCCTGCCTGACCACGTGAGCATTGTGGAACCCAAAGATGAGATACTGCCCACCACCCCCATCTCAGAACAGAAGGGTGGGAAGCCAGAGCCGCCTGCCATGCCCCAGCCAGTCCCCACAGCACTCGAGCACCACCACCACCACCACTGA-3’ |

| GST-SEC14L3 |
| --- |
| 5’-ATGTCCCCTATACTAGGTTATTGGAAAATTAAGGGCCTTGTGCAACCCACTCGACTTCTTTTGGAATATCTTGAAGAAAAATATGAAGAGCATTTGTATGAGCGCGATGAAGGTGATAAATGGCGAAACAAAAAGTTTGAATTGGGTTTGGAGTTTCCCAATCTTCCTTATTATATTGATGGTGATGTTAAATTAACACAGTCTATGGCCATCATACGTTATATAGCTGACAAGCACAACATGTTGGGTGGTTGTCCAAAAGAGCGTGCAGAGATTTCAATGCTTGAAGGAGCGGTTTTGGATATTAGATACGGTGTTTCGAGAATTGCATATAGTAAAGACTTTGAAACTCTCAAAGTTGATTTTCTTAGCAAGCTACCTGAAATGCTGAAAATGTTCGAAGATCGTTTATGTCATAAAACATATTTAAATGGTGATCATGTAACCCATCCTGACTTCATGTTGTATGACGCTCTTGATGTTGTTTTATACATGGACCCAATGTGCCTGGATGCGTTCCCAAAATTAGTTTGTTTTAAAAAACGTATTGAAGCTATCCCACAAATTGATAAGTACTTGAAATCCAGCAAGTATATAGCATGGCCTTTGCAGGGCTGGCAAGCCACGTTTGGTGGTGGCGACCATCCTCCAAAATCGGATCTGGTTCCGCGTGGATCCCCGGAATTCATGAGCGGCCGAGTTGGAGACCTGAGCCCCAAACAGGCAGAGACCCTGGCCAAGTTCCGAGAAAACGTCCAGGATGTGCTTCCTGCCCTGCCCAACCCTGATGATTATTTCCTTCTACGCTGGCTCCGAGCTCGGAATTTTGACTTGCAGAAGTCGGAGGCTTTGCTCCGCAAGTACATGGAGTTCCGGAAGACCATGGATATTGACCATATCCTTGATTGGCAGCCCCCAGAGGTGATCCAGAAGTACATGCCTGGGGGCCTGTGTGGCTATGACCGTGATGGCTGCCCCGTGTGGTATGACATCATTGGGCCACTTGATCCCAAGGGGTTGCTCTTCTCAGTCACCAAGCAGGACCTGCTCAAGACCAAGATGAGGGACTGTGAGCGCATCCTGCATGAGTGTGACCTGCAGACAGAGAGGCTAGGGAAGAAGATTGAGACCATCGTGATGATATTTGACTGTGAGGGCCTGGGACTGAAACACTTCTGGAAACCTCTGGTAGAAGTGTACCAGGAGTTCTTTGGCCTCCTTGAAGAGAATTACCCAGAGACCCTGAAGTTCATGCTCATCGTGAAAGCTACCAAACTGTTCCCTGTGGGCTACAACCTCATGAAGCCATTCCTGAGTGAGGACACTCGCAGGAAAATTATTGTGTTGGGAAATAACTGGAAGGAAGGTTTGCTGAAACTCATCAGTCCTGAGGAACTGCCTGCCCAGTTTGGGGGCACCCTGACTGACCCAGATGGGAACCCCAAATGTTTAACCAAGATTAACTATGGCGGGGAGATCCCCAAGTCCATGTACGTGCGGGACCAGGTGAAGACTCAGTACGAGCACTCGGTGCAGATCAACCGCGGCTCATCACACCAAGTGGAATACGAGATCCTATTTCCAGGCTGCGTTCTCAGGTGGCAGTTCTCATCTGATGGTGCGGACATCGGCTTCGGAGTTTTCCTGAAGACCAAGATGGGGGAGCGACAGCGGGCAGGGGAGATGACAGATGTTCTACCCAGCCAGCGCTATAACGCCCACATGGTGCCCGAGGATGGGAACCTCACCTGCTCAGAGGCCGGCGTCTATGTCCTACGCTTCGACAACACCTATAGCTTTGTCCACGCCAAGAAGGTCAGCTTCACAGTGGAGGTCCTGCTCCCTGACGAGGGCATGCAGAAATATGATAAGGAGCTCACCCCTGTCTAG-3’ |
