# Supplementary material for: SEC14L3 knockdown inhibited clear cell renal cell carcinoma proliferation, metastasis and sunitinib resistance through an SEC14L3/RPS3/NFκB positive feedback loop
Source: J Exp Clin Cancer Res. 2024 Oct 19;43:288. doi: 10.1186/s13046-024-03206-5 (PMC11490128; doi:10.1186/s13046-024-03206-5)
Supplement: Supplementary file 9 — Supplementary Material 9 [file 13046_2024_3206_MOESM9_ESM.docx]

| Patient number | Name | Gender | Age | Pathological type | T | N | M | isup |
| --- | --- | --- | --- | --- | --- | --- | --- | --- |
| F07684 | Xuewu Ji | M | 62 | Clear cell renal cell carcinoma | 1 | 0 | 0 | 2 |
| F09862 | Zuyin Mai | M | 73 | Clear cell renal cell carcinoma | 2 | 1 | 0 | 4 |
| F09965 | Guocai Chen | M | 65 | Clear cell renal cell carcinoma | 1 | 0 | 0 | 2 |
| F12370 | Zhengsu Yan | M | 70 | Clear cell renal cell carcinoma | 1 | 0 | 0 | 2 |
| F16400 | Qinkang Shen | W | 74 | Clear cell renal cell carcinoma | 1 | 0 | 0 | 2 |
| F31173 | Xujie Wu | W | 84 | Clear cell renal cell carcinoma | 2 | 1 | 0 | 4 |
| F27837 | Jianhua Lu | M | 62 | Clear cell renal cell carcinoma | 1 | 0 | 0 | 2 |
| F19955 | Weiqi Li | W | 72 | Clear cell renal cell carcinoma | 1 | 0 | 0 | 2 |
| F22171 | Zhenming Yao | M | 63 | Clear cell renal cell carcinoma | 1 | 0 | 0 | 2 |
| F27089 | Fuming Xu | M | 62 | Clear cell renal cell carcinoma | 1 | 0 | 0 | 2 |
| F34337 | Genyuan Mao | M | 67 | Clear cell renal cell carcinoma | 1 | 0 | 0 | 2 |
| F33237 | Jinhua Zhu | M | 64 | Clear cell renal cell carcinoma | 1 | 0 | 0 | 2 |
