# Supplementary material for: SEC14L3 knockdown inhibited clear cell renal cell carcinoma proliferation, metastasis and sunitinib resistance through an SEC14L3/RPS3/NFκB positive feedback loop
Source: J Exp Clin Cancer Res. 2024 Oct 19;43:288. doi: 10.1186/s13046-024-03206-5 (PMC11490128; doi:10.1186/s13046-024-03206-5)
Supplement: Supplementary file 10 — Supplementary Material 10 [file 13046_2024_3206_MOESM10_ESM.docx]

**Fig S1. SEC14L3 is overexpressed in ccRCC.** **a, b.** GEO database analyses of the SEC14L3 expression in ccRCC tumor samples (**a**) and overall survival curve of ccRCC patients with low and high SEC14L3 expression (**b**). **c.** Western blot analysis of SEC14L3 expression levels in 12 ccRCC tissues and paired adjacent tissues.

**Fig S2. siRNA-mediated knockdown of SEC14L3 inhibits ccRCC cell proliferation, migration and invasion in vitro.** **a, b.** Western blot (**a**) and qRT‒PCR (**b**) analyses were performed to examine the knockdown of SEC14L3 in 786-O cells mediated by siRNA infection. **c-f.** CCK-8 (**c**), colony formation (**d, e**) and EdU (**f, g**) assays were performed to evaluate the proliferation capacity of 786-O SI1, 2 cells. **h-l.** wound healing assays (**h, i**) and transwell assays **(j-l**) were employed to evaluate the cell migration and invasion capabilities of 786-O SI1, 2 cells. The data are presented as the means ± SDs of three independent experiments. *p < 0.05, **p < 0.01, ***p < 0.001, ****p < 0.0001.

**Fig S3. Lentiviral knockdown of SEC14L3 inhibits ccRCC cell proliferation, migration and invasion in vitro.** **a-g.** The statistical histograms of Western blot (**a**), qRT‒PCR (**b**), Colony formation (**c**), EdU wound healing and transwell assays for comparing differences between the NC and KD groups. The data are presented as the means ± SDs of three independent experiments. *p < 0.05, **p < 0.01, ***p < 0.001, ****p < 0.0001.

**Fig S4. SEC14L3 knockdown inhibits NFκB nuclear translocation and inactivates the NF-κB signaling pathway in ccRCC. a.** Based on RNA-seq analysis, KEGG analysis revealed a close association between SEC14L3 and the NFκB signaling pathway. **b, c**. Western blot analysis of NFKB1 expression levels in 786-O and A-498 KD cells. The data are presented as the means ± SDs of three independent experiments. *p < 0.05, ***p < 0.001.

**Fig S5. SEC14L3 interacts with RPS3 and negatively regulates its protein level through ubiquitination. a, b.** His-RPS3 (**a**) and GST-SEC14L3 (**b**) proteins, expressed in an E. coli system, were detected by SDS-PAGE. **c, d.** Western blot analysis was performed to demonstrate that CQ treatment (25μM, 24h) had no significant effect on RPS3 protein levels in 786-O KD and A-498 KD cells. **e, f.** Western blot analysis was performed to demonstrate that MG132 treatment (10μM,8h) rescued RPS3 protein levels in 786-O KD and A-498 KD cells. The data are presented as the means ± SDs of three independent experiments. *p < 0.05, **p < 0.01, ***p < 0.001.

**Fig S6. The NPs could be phagocytosed by ccRCC cells and reduce SEC14L3 expression. a.** Images of NPs-mediated cytophagocytosis in 786-O cells. **b-d.** Western blot (**b, c**) and qRT‒PCR (**d**) analyses were performed to determine whether the knockdown of SEC14L3 in 786-O cells was mediated by NP phagocytosis. **e-i.** CCK-8 (**e**), Colony formation (**f, g**) and EdU (**h, i**) assays were performed to evaluate the proliferative capacity of 786-O cells phagocytosed with NPs. **j-n.** Transwell assays (**j-l**) and wound healing assays (**m, n**) were employed to evaluate the cell migration and invasion capabilities of NPs phagocytosed 786-O cells. The data are presented as the means ± SDs of three independent experiments. *p < 0.05, **p < 0.01, ***p < 0.001, ****p < 0.0001.

**Fig S7. The biosafety of the NPs. a.** Serum analysis of BUN, creatinine, ALT and AST levels in mice following intravenous injection of NPs. **b.** H&E staining of lung, liver, spleen, kidney and heart tissues from mice treated with NPs. NS not significant.
